# A minimum information standard for reproducing bench-scale bacterial cell growth and productivity

**DOI:** 10.1038/s42003-018-0220-6

**Published:** 2018-12-06

**Authors:** Ariel Hecht, James Filliben, Sarah A. Munro, Marc Salit

**Affiliations:** 1 Joint Initiative for Metrology in Biology, 443 Via Ortega, Room 325, Stanford, CA 94305 USA; 2 Genome-scale Measurements Group, National Institute of Standards and Technology, 443 Via Ortega, Room 325, Stanford, CA 94305 USA; 3 Department of Bioengineering, Stanford University, 443 Via Ortega, Room 325, Stanford, CA 94035 USA; 4 Statistical Engineering Division, 100 Bureau Drive, National Institute of Standards and Technology, Gaithersburg, MD 20899 USA; 5 SLAC National Accelerator Laboratory, Menlo Park, CA 94025 USA; 6 Present Address: Minnesota Supercomputing Institute, University of Minnesota, Minneapolis, Minnesota, 55455 USA

**Keywords:** Metabolic engineering, Metabolic engineering

## Abstract

Reproducing, exchanging, comparing, and building on each other’s work is foundational to technological advances. Advancing biotechnology calls for reliable reuse of engineered organisms. Reliable reuse of engineered organisms requires reproducible growth and productivity. Here, we identify the experimental factors that have the greatest effect on the growth and productivity of our engineered organisms in order to demonstrate reproducibility for biotechnology. We present a draft of a Minimum Information Standard for Engineered Organism Experiments (MIEO) based on this method. We evaluate the effect of 22 factors on *Escherichia coli* engineered to produce the small molecule lycopene, and 18 factors on *E. coli* engineered to produce red fluorescent protein. Container geometry and shaking have the greatest effect on product titer and yield. We reproduce our results under two different conditions of reproducibility: conditions of use (different fractional factorial experiments), and time (48 biological replicates performed on 12 different days over 4 months).

## Abstract (web summary)

Ariel Hecht et al. have developed a minimum information standard for use in biotechnology. They show the most important factors in maintaining reproducibility in handling engineered organisms under different experimental conditions.

## Introduction

The irreproducibility of experimental results in biotechnology^1^ and bioengineering^2^ must be overcome to realize the potential of biology as a reliable engineering substrate^3,4^. The synthetic biology community has expressed a desire for experimental protocol standards^5–7^, supplementing existing standards for genetic modifications^8^. Minimum information standards have improved reproducibility for qPCR^9^, microarray^10^, and genomics^11^ experiments, and a minimum information standard could similarly improve the reproducibility of engineered cell experiments. There have been calls to address reproducibility with reference strains^5,7^. While reference strains and information standards can and should coexist, information standards are more generalizable, accessible, verifiable, and maintainable. There have also been several efforts to improve reproducibility through software and automation^12–15^.

Biological engineering typically proceeds in three steps: genetically modifying the organism, growing the organism, and assaying its function (Supplementary Fig. 1). The conditions under which engineered cells are grown can have a large impact on the cell’s performance—the relationship between a genetic modification and its function cannot be fully defined without considering the growth conditions. Here, we describe a method to systematically evaluate the effect of experimental factors on growth/productivity of engineered cells, and will recommend the development of a minimum information standard based on this method.

We hypothesize that a sufficient description of experimental factors will enable reproducible performance of engineered cells, and that we can realize this description by building a literature knowledgebase to identify factors, measuring factor effects with an appropriate orthogonal factorial experimental design, and demonstrating that controlling these factors results in reproducible growth and productivity (see Methods section for definitions of repeatability and reproducibility). This description can form the basis of a minimum information standard for growth/productivity of engineered organisms. We will test our hypothesis with two test cases using publicly available strains, *Escherichia coli* engineered to produce the small molecule lycopene, and the heterologous protein RFP.

This paper focuses on experimental factors that define and influence growth conditions for engineered cells. Genetic modifications, both intentional and those arising from evolution, are outside of our scope. Cellular assays are also outside of our scope. We focus on experimental factors at bench scale (microtiter plates and shake flasks) in batch culture mode because these formats are often the first step in most bioengineering projects, and unlike larger fermenters, microtiter plates and shake flasks do not allow for continuous monitoring and control of many factors.

This paper demonstrates that fractional factorial designs can be used to identify the experimental factors that have the greatest effect on the growth and productivity of engineered organisms. In our two test cases, we found that the geometry and shaking of the growth container have the largest effects on the growth and productivity of our organisms, and that these factor effects are reproducible. We found that we can achieve reproducible performance by carefully controlling these factors. We used these data to develop a Minimum Information Standard for Engineered Organisms Experiments (MIEO) that will help improve the repeatability and reproducibility of engineered organism experiments.

## Results

### Experimental factors and experimental design

The factors that affect cell growth/productivity of engineered *E. coli* can be grouped into three broad categories: media; container, which is the culture vessel within which cells are grown, such as microtiter plates or shake flasks; and other factors, including time, environment, selective agents, and inoculum (Supplementary Fig. 1). We identified 32 experimental factors that have been reported to affect cell growth/productivity (Table 1).

**Table 1:** 32 experimental factors that have been documented to affect cell growth and productivity

	Factor	References	Experimental factor	Low	Center	High
**Media**	*Components*					
	Media type	^39– 47^				
	Carbon source	^41, 45, 47– 51^	Yeast extract (g L^-1^)	20	24	28
			Glycerol (g L^-1^)	3	5	7
	Nitrogen source	^47, 52^	Tryptone (g L^-1^)	10	12	14
	Inorganic ions	^18, 52, 53^	Magnesium sulfate (g L^-1^)	0	0.12	0.24
	Manufacturer	^18^	Yeast extract source	Sigma	---	Millipore
	*Properties*					
	pH	^52, 54– 58^	pH	6.7	7.2	7.5
			Buffer capacity (mmol L^-1^)	70	90	110
	Osmolality	^30, 52, 58, 59^	Osmolality (mmol kg^-1^)	650	750	850
	Media viscosity	^60– 63^				
**Container**	*Geometry*					
	Volume	^21, 61, 64^	Well volume (mL)	2.5	---	10
			Flask volume (mL)	125	---	250
	Fill volume	^21, 44, 46, 61, 65– 69^	Well fill volume	10%	---	30%
			Flask fill volume	7.5%	---	15%
	Cover	^68, 70– 74^	Well cover	Aeraseal	---	Aluminum
			Flask cover	Foam	---	Aluminum
	Well shape	^44, 64, 66, 67^				
	Well bottom	^44^	Well bottom	Round	---	Pyramidal
	Flask baffles	^60, 61, 73– 78^	Flask baffles	Unbaffled	---	Baffled
	Baffle geometry	^61^				
	Surface coating	^67, 79^				
	Container diameter	^46, 55^				
	*Shaking*					
	Shake speed	^21, 44– 46, 61, 64– 68, 70, 79– 81^	Shake speed (rpm)	230	---	460
	Shake diameter	^46, 64, 65, 67, 79, 80^				
**Other**	*Time*					
	Time	^40, 44^	Time (h)	24	48	72
	*Environment*					
	Temperature	^39, 40, 44, 58, 82^	Temperature (°C)	30	---	37
	Incubator O_2_ concentration	^41^				
	Lab	^2, 51, 83– 85^				
	Operator	^85^				
	*Inoculum*					
	Inoculum concentration	^44, 86^	Inoculum amount (cells mL^-1^)	2 x 10^6^	8 x 10^6^	3 x 10^7^
			Inoculum age (h)	3	16	16 + 96
	Glycerol in freezer stock	^87^				
	Cell phase at preservation	^87^				
	Time in storage	^88^				
	*Selective agents*					
	Antibiotic type	^51^				
	Antibiotic concentration	^39, 89^	Antibiotic concentration (μg mL^-1^)	6.25	25	100
	*Inducers*					
	Inducer amount	^40, 44, 90, 91^				
	Induction time	^44, 90^				

The factors are grouped in three broad categories, and can be further broken down to 9 more specific categories. From this list, we selected 22 factors that were practically accessible to us in the laboratory to evaluate. For each factor, we selected two levels, low and high, for evaluation in a fractional factorial design. For quantitative factors, where possible, we added a third center level.

We evaluated these factors with orthogonal two-level fractional and full factorial experiments. Factorial designs have two main advantages compared with evaluating one factor at a time: increased precision in estimating factor effects with minimal bias from factor interactions, and the ability to detect interactions between multiple factors. Factorial designs also have some limitations: estimates of factors are limited to the levels selected for each factor^16^.

For our first test case, *E. coli* engineered to constitutively produce the small molecule lycopene^17^, we evaluated the effect of 22 factors on three responses: dry cell mass, titer, and yield (yield is the ratio of titer to dry cell mass, and is a dimensionless, scalable parameter). We chose these 22 factors because they were accessible in our laboratory and relevant to our test strain. For each factor, we selected two levels, low and high; a center level was included when possible (Table 1). We quantified the effect of each factor on the responses by the relative effect magnitude, which is the absolute value of the difference between the mean response at the two levels divided by the overall mean response.

**Fig. 1:**
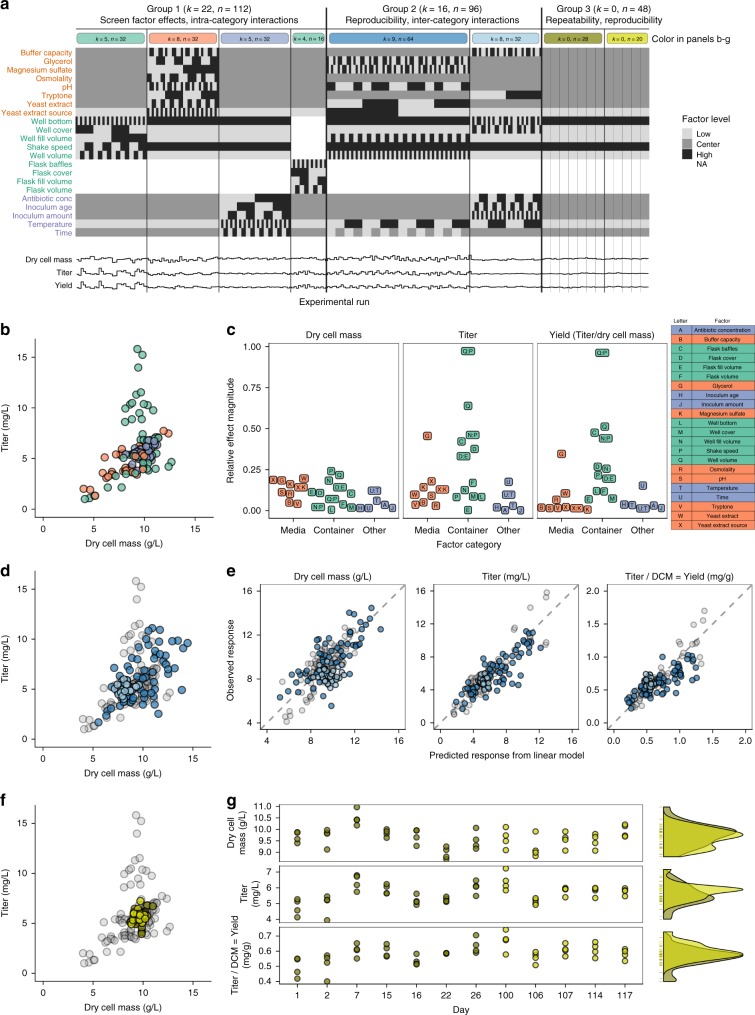
Repeatability and reproducibility of factor effects on cell growth and lycopene productivity. **a** Experimental design table with 22 factor rows, and 256 experimental run columns. Colored bars at the top correspond to color of points in other panels. *k* is the number of factors varied, and *n* is the number of runs, in a group or experiment. The three responses, normalized to range from 0 to 1, are given below. **b** Dynamic range of lycopene titer and dry cell mass observed in Group 1 experiments. **c** Relative effect magnitude of all 22 factors on all three responses, colored by factor category. **d** Dynamic range of lycopene titer and dry cell mass observed in Group 2 experiments. **e** Factor effects observed in Group 1 are reproducible in Group 2. Gray points are Group 1 data used to train a linear model (dry cell mass r^2^ = 0.66, titer r^2^ = 0.85, and yield r^2^ = 0.87). Blue points are Group 2 used to test a linear model (dry cell mass r^2^ = 0.46, titer r^2^ = 0.71, and yield r^2^ = 0.56). **f** Dynamic range of titer and dry cell mass observed in Group 3 centerpoint replicates. **g** Centerpoint replicates plotted as a function of day on which they were run show no trends over time (left). Density plots (smoothed histograms) of response distribution in the first 26 days (dark yellow) and last 17 days (light yellow) overlap

Our experimental design consisted of 256 experimental runs organized into three groups. A run is a single combination of experimental growth conditions. Runs were grouped to answer a particular question: which factors have the largest effect on growth/productivity (Group 1), are those effects reproducible under different conditions of use (Group 2), and is a single set of conditions repeatable and reproducible over time (Group 3, see Methods for definitions of repeatability and reproducibility). Because it was logistically impossible for us to execute all of the runs in a group in one experiment, groups were divided by factor category (Group 1), factor effect (Group 2), and time (Group 3) for execution in sequential factorial experiments (Fig. 1a and Supplementary Data 1).

### Lycopene test case

Group 1 screened factor effects and intra-category interactions in 112 runs, divided into four experiments by factor category: media composition, microwell containers, shake flask containers, and other, using 2^(8–3)^, 2^5^, 2^4^, and 2^5^ orthogonal factorial designs, respectively, with appropriate randomization. These designs allowed us to estimate the main effect of each factor and to detect two-factor interactions within each category. These designs did not allow us to detect interactions between factors in different categories, which we addressed in Group 2.

Varying the factor levels for growing our strain  resulted in a dynamic range of 4–12 g L^−1^ for dry cell mass and 1–16 mg L^−1^ for lycopene titer (Fig. 1b and Supplementary Fig. 2). We calculated the relative effect magnitude of two-factor interactions for all pairs of factors in each experiment (Supplementary File 1b), and identified the largest two-factor interactions by examining a normal probability plot of the effects (Supplementary Fig. 3). Particularly interesting two-factor interactions (Q:P, N:P, X:K) occurred between yeast extract source and magnesium sulfate (supplementing the media with 0.24 g L^−1^ magnesium sulfate^18^ eliminated the effect of yeast extract source) and between container factors (nonlinear interactions between shaking speed, container volume, and fill volume) (Supplementary Figs. 4–6).

Container factors and glycerol had the largest effect on strain productivity (Fig. 1c and Supplementary Fig. 7). Container factors primarily affect oxygen transfer into the media, but can also affect exchange of other gases, shear forces, and mixing within the media^19–21]^. The biggest single effect was the interaction between well volume and shake speed. Titer was more sensitive to container factors than dry cell mass. Dry cell mass and titer were equally sensitive to media factors. Except for glycerol, yield was not sensitive to media factors—changing the media composition affected the total amount of cells that grew in the culture, but not the per-cell productivity. Time and temperature had relatively small effects at the levels used here.

In Group 2, we evaluated the reproducibility of factor effects and screened inter-category interactions in 96 runs. We split the factors into two experiments based on the results from Group 1: nine factors that had large effects on the responses, and eight factors that had small effects on the responses, using 2^(9–3)^ and 2^(8–3)^ orthogonal fractional factorial designs, respectively (Fig. 1d). We trained a linear model with the Group 1 data to predict responses from these factors (Supplementary Note 1 and Supplementary Data 1). We then tested this model by using it to predict the responses in Group 2 (Fig. 1e). These results show that the factor effects are predictable by a linear model, reproducible under different conditions of use, and that there were no measureable two-factor interactions between factors in different categories (Supplementary Fig. 8 and Data 1d).

In Group 3, we evaluated the repeatability and reproducibility of the growth/productivity at a single set of factor levels (centerpoint levels) in 48 runs. Four replicate runs were performed on each of 12 different days over 4 months: 7 days in the first month and 5 days in the fourth month (Fig. 1f, g). The variance within each of the 12 days was homogeneous (Supplementary Table 1). The repeatability within a single day (mean repeatability standard deviation (SD)) was 3.5% for dry cell mass, 7.2% for titer, and 8.2% for yield. The reproducibility between the first and fourth month (reproducibility SD) was 4.9% for dry cell mass, 11.4% for titer, and 11.4% for yield. The distribution of the data from the two months was similar, with month only accounting for 2.3% (dry cell mass), 4.9% (titer), and 8.7% (yield) of the variance with the population, as determined by an analysis of variance (Fig. 1g). These results show that using our method to identify and control experimental parameters allowed us to reproduce our results over time.

### RFP test case

For our second test case, we evaluated the effect of 18 factors on the dry cell mass, titer, and yield of *E*. *coli* BW25113 engineered to constitutively express the heterologous protein RFP^22^. We did not include the four shake flask factors, as these were not accessible in our laboratory for this test case. We observed similar results as with lycopene, except for different relative effect magnitudes of the container factors on titer (Supplementary Figs. 9–16 and Supplementary Data 2). We speculate that these differences may be due to differences in the utilization of oxygen in the biosynthetic pathways of the two products—lycopene is derived from central metabolism, and RFP is a heterologous protein.

## Discussion

We have determined a sufficient description of experimental factors that enabled repeatable and reproducible measurements of growth and productivity of two engineered *E. coli* strains. This demonstrates proof-of-principle of our approach, and is a step toward the creation of a minimum information standard for growth conditions of engineered organisms, which would support interoperability of engineered parts and enable assessment of reproducibility^5,23^.

Experimental growth conditions are frequently specified and documented by free-form text, such as the methods sections of most journals. These unstructured narratives are problematic because it is left to the authors to decide what information to include, and they can be difficult to parse. We propose developing a Minimum Information Standard for Engineered Organism Experiments (MIEO), using a method such as that described here, to address this issue. MIEO should be useful for any biological engineer who is planning experiments, reporting results, comparing results, or reproducing results within and between organizations.

To maximize the success of MIEO, we will incorporate lessons about modularity and simplicity learned from previous minimum information checklists. MIEO will be a modular checklist^11,24,25^, capturing information in nine categories (Table 2). The factors that should be included in each category will be different in each experiment (Supplementary Note 2). Categories will be designated as required or optional for reporting, similar to other standards^9,11^. Categories are optional because they can be derived from other categories, or are not applicable in every situation. MIEO is intended to be compatible with any cell type, and any downstream assay, complementing the existing suite of minimum information checklists^25^.

**Table 2:** Minimum Information Standard for Engineered Organism Experiments (MIEO) v0.1, with Group 3 factors and levels as an example (Fig. 1a)

MIEO category	Factor	Level
Media components (R)	Yeast extract	24 g L^−1^
	Yeast extract source	Sigma Y1625-250G, Lot SLBR9838V
	Glycerol	5 g L^−1^
	Magnesium sulfate	0.12 g L^−1^
	Tryptone	12 g L^−1^
	Potassium phosphate	2.28 g L^−1^
	Dipotassium phosphate	12.7 g L^−1^
	Sodium chloride	6.63 g L^−1^
	Water	DI water (18 MΩ-cm)
Media properties (O)	pH	7.2
	Buffer capacity	90 mmol L^−1^
	Osmolality	750 mmol kg^−1^
Container geometry (R)	Type	96-well plate
	Well shape	Square
	Well bottom	Round
	Well volume	2.5 mL
	Fill volume	10% (0.25 mL)
	Cover	AeraSeal
Container shaking (R)	Shaking speed	460 rpm
	Shaking diameter	12.5 mm
	Shaking mode	Orbital
Time (R)	Growth time	48 h
Environment (R)	Temperature	30 °C
	Relative humidity	80%
Selective agents (O)	Antibiotic type	Chloramphenicol
	Antibiotic concentration	25 μg mL^−1^
Inoculum (R)	Concentration at inoculation	OD_600_ = 0.01
	Age of inoculum at inoculation	16 h
Inducers (O)	None	None

The nine MIEO factor categories are given, along with whether their reporting is required (R) or optional (O). Factors and their level are reported

One of the key factors that determine the adoption of a minimum information standard is simplicity^26^. We will aim for simplicity by limiting the standard to nine categories of information, and by including both human-readable/writable (e.g., a table created in word-processing or spreadsheet software) and machine-readable/writable (e.g., XML) implementations. While a machine-readable/writable format has obvious appeal, the cost of adopting such a standard can be prohibitive for some. We have created an example checklist based on the centerpoint experimental conditions used in this paper (Table 2).

Standards development is best as a community-driven bottom-up effort, not a top–down prescription^27,28^. We encourage other members of the biotechnology community to contribute to the development of this standard (http://jimb.stanford.edu/mieo/).

In our experiments, we found that the geometry and shaking of the growth container had the largest effect on productivity. These factors warrant special consideration, because they are often tied to large capital expenditures, and can be difficult to change. Given their nonlinear effects, if factor levels cannot be matched in different labs, then expectations about reproducibility should be adjusted accordingly.

The challenges facing the reproducibility of experimental data in biology are momentous. The results shown in this paper demonstrate a method for reproducing key experimental results over time and under different conditions of use. A well-implemented and widely adopted minimum information standard would improve the repeatability and reproducibility of engineered organism experiments. Experimental reproducibility would advance biological engineering toward becoming a more reliable and predictable engineering discipline.

## Methods

### Strain engineering

The parent strain for both test strains used in this paper was *Escherichia coli* BW25113 (Δ(*araD-araB)567 ΔlacZ4787(:rrnB-3) λ*^*−*^ *rph-1 Δ(rhaD-rhaB) 568 hsdR514)* obtained from the Yale Coli Genetic Stock Center (New Haven, CT). For production of lycopene, the parent strain was transformed with plasmid pAC-LYC^17^, a gift from Francis X. Cunningham Jr (Addgene plasmid #53270). For production of RFP (mRFP1), the parent strain was transformed with plasmid pFAB3992^22^, a gift from Drew Endy (Addgene plasmid #47823). Plasmid sequences (Supplementary Data 3) and maps (Supplementary Fig. 17) are available online. All reagents used in this paper with manufacturer, product, and lot numbers are available online (Supplementary Table 2).

pAC-LYC was received transformed in *E. coli* Top10 in an agar stab (Addgene plasmid #53270). A plate of lysogeny broth (LB) agar (10 g L^−1^ tryptone, 5 g L^−1^ yeast extract, 10 g L^−1^ sodium chloride, and 15 g L^−1^ bacto agar) with 25 μg mL^−1^ chloramphenicol was streaked with a sterile pipette tip dipped into the agar stab, and incubated overnight at 37 °C. A single colony was picked, and grown overnight in 5 mL of LB (10 g L^−1^ tryptone, 5 g L^−1^ yeast extract, and 10 g L^−1^ sodium chloride) with 25 μg mL^−1^ chloramphenicol. The plasmid was prepared using the manufacturer’s provided protocol. Plasmid identity was confirmed by digestion with Pst1-HF (Supplementary Fig. 18).

The parent strain was received on a dehydrated paper disk. The disk was placed on an LB agar plate using sterilized forceps, and rehydrated with one drop of LB. A sterile pipette tip was used to streak the moisture from the disk, and the plate was incubated overnight at 37 °C. A single colony was carefully picked with a sterile pipette tip and inoculated into 5 mL of LB, which was incubated overnight for 16 h at 37 °C, shaking at 250 rpm with a 25 mm shaking diameter (Thermo Fisher Forma Model 440 Orbital Shaker). In total, 20 μL of the overnight culture was used to inoculate 2 mL of fresh LB, which was then incubated in the same incubator for 2 h. The cells were resuspended in 100 μL of 100 mM of ice-cold sterile-filtered calcium chloride solution in a 1.5 mL microcentrifuge tube. In total, 1 μL of pAC-LYC prepared plasmid (approximate concentration 90 ng μL^−1^) was added to the cell suspension, and the cells were incubated on ice for 30 min. The cells were heat-shocked in a 42 °C water bath for 30 s, and then incubated on ice for 2 min. In total, 400 μL of SOC media was added to the cells, and they were incubated at 37 °C with gentle mixing for 1 h. In total, 25 μL of the cell solution was pipetted onto an LB agar plate with chloramphenicol and incubated overnight at 37 °C. A single colony was picked from the agar plate, incubated in 5 mL of LB with chloramphenicol, and grown for 4 h. In total, 750 μL of the culture was mixed with 750 μL of 50% sterile-filtered glycerol in a cryovial for long-term storage at −80 °C. All lycopene-producing cells used in this paper were derived from this glycerol stock.

pFAB3992 was received transformed in *E*. *coli* BW25113 in a frozen glycerol stock. A plate of LB agar with 50 μg mL^−1^ kanamycin was streaked with a sterile pipette tip streaked across the glycerol stock, and incubated overnight at 37 °C. A single colony was picked from the agar plate, incubated in 5 mL of LB with kanamycin, and grown for 4 h. In total, 750 μL of the culture was mixed with 750 μL of 50% sterile-filtered glycerol in a cryovial for long-term storage at −80 °C. All RFP-producing cells used in this paper were derived from this glycerol stock.

### Cell culture

LB agar plates with the appropriate antibiotic (25 μg mL^−1^ chloramphenicol or 50 μg mL^−1^ kanamycin) were streaked with a sterile pipette tip from glycerol stocks, and incubated overnight at 37 °C. Plates were stored, wrapped in Parafilm M (Bemis NA, Neenah WI), at 4 °C for up to 2 weeks, after which they were discarded. All cultures were grown in variants of Terrific Broth (TB), which has a baseline composition of 24 g L^−1^ yeast extract, 12 g L^−1^ tryptone, 5 g L^−1^ glycerol, 0.17 mol L^−1^ KH_2_PO_4_, and 0.72 mol L^−1^ K_2_HPO_4_^29^. These values were used as centerpoint values for media composition. We modified this recipe by adding magnesium sulfate to supplement deficient magnesium content in the yeast extract^18^, and adding sodium chloride to adjust the osmolality^30^.

Every experiment began with a liquid starter culture. A single colony was picked from the agar plate, inoculated into 4 mL of TB with appropriate antibiotic in a plastic-capped 16 × 100-mm glass culture tube (VWR 47729-576), and grown for 16 h at 37 °C, shaking at 250 rpm with a 25-mm shaking diameter. This culture was diluted with phosphate-buffered saline (PBS) to an OD_600_ = 0.5 (as measured in a BRAND semi-micro polystyrene cuvette on a WPA Biowave CO8000 Cell Density Meter), and was then used as the starter culture for inoculating the experimental runs. A fresh starter culture was prepared on each day on which an experimental run was started. All experimental runs started on the same day were inoculated from the same starter culture, except for cultures with different inoculum age. For the inoculum age of 3 h, a 50 μL aliquot of the 16 h starter culture was taken and used to inoculate 4 mL of fresh media in a glass culture tube, and returned to the incubator for 3 h, then removed, and diluted to OD_600_ = 0.5. This culture was in exponential phase at the time that it was removed from the incubator. For the inoculum age of 16 + 96 h, after the usual 16-h incubation, the starter culture was stored at 4 °C for 96 h, and then removed and diluted to OD_600_ = 0.5 for use.

For cultures with varying pH or varying buffer capacity, media composition was determined by simultaneously solving the Henderson–Hasselbach equation:

1 $${\mathrm{pH}} = {\mathrm{pKa}} + {\mathrm{log}}_{10}\left( {\frac{{\left[ {A^ - } \right]}}{{\left[ {HA} \right]}}} \right)$$

and the equation for buffer capacity:

2 $${\mathrm{Buffer}}\,{\mathrm{capacity}} = \left[ {A^ - } \right] + \left[ {HA} \right]$$

for *[A*^*−*^*]* and *[HA]*, where *A*^*−*^ is the conjugate base, *HA* is the conjugate acid, and pKa for phosphate buffers is 6.86^31^. Solving these two equations for the baseline TB composition gives pH = 7.5 and buffer capacity = 90 mM. Adding the remaining media components lowered the pH by 0.3–0.5 pH units. In designing the experiments, we aimed for pH of 7.0, 7.5, or 8.0. We measured the pH of the media with a pH meter, and found that the actual pH was ~6.7, 7.2, or 7.5, respectively.

For cultures with varying osmolality, the osmolality of all the media components was calculated assuming complete dissociation of ionic species, and the empirically determined osmolality of yeast extract and tryptone of 6 mmol L kg^−1^ g^−1^. Sodium chloride was added to increase the osmolality to the desired level^30^. The osmolality of 12 different media solutions, as measured by the Wescor Vapro 5520 Vapor Pressure Osmometer, was within 7% of the target value (Supplementary Table 3).

The growth of our test strains for the factorial experiments (Fig. 1a and Supplementary Fig. 9a) was performed under the conditions listed in Table 1 and Supplementary Data 1-2, and the growth of our test strains for the centerpoint replicates was performed under the conditions listed in Table 2. The full experimental design table, with all settings for each run, is available online (Supplementary Data 1-2). Stocks of each individual media component were prepared at 10X concentration of the centerpoint value with DI water except for magnesium sulfate and dipotassium phosphate, which were purchased. Stocks were sterilized by an autoclave except for glycerol, which was sterilized by a 0.22 μm syringe filter (Whatman Puradisc FP30). The position of each run on a plate, and the order in which the runs were set up and sampled, was randomized.

Four different types of container covers were used (Supplementary Table 2 and Data 1, 2). Microwell plates were covered with either the gas-permeable, adhesive AeraSeal membrane (E&K Scientific), or the AeraSeal membrane and a gas-impermeable aluminum foil tape (Bio-Rad). The AeraSeal membrane was first applied to prevent direct contact between the liquid media and the aluminum foil tape. Shake flasks were covered with either the gas-permeable silicone foam covers (Bellco Glass), or two layers of gas-impermeable aluminum foil secured with ParaFilm wrapped around the base of the neck of the flask.

### Assay calibration

Our absorbance assay for lycopene was calibrated using a 1mg lycopene standard (Sigma, L9879-1MG, Lot# SLBS4759 and SLBV5371). The lyophilized standard was stable for only 48 h after being dissolved in solvent. A 1:1:1 (v/v/v) mixture of methanol, acetone, and dichloromethane was the solvent for the calibration. The mixture was prepared by mixing 30 mL each of the three solvents. The lycopene standard was dissolved in 40 mL of solvent in a 50 mL polypropylene Falcon tube by shaking and vortexing for 10 min until the standard dissolved. The tube was covered with aluminum foil to minimize exposure to light. In total, 240 μL of the standard solution was diluted with 2.76 mL of solvent to create a solution with nominal concentration 2 μg mL^−1^. This solution was transferred to a Hellma Analytics 6030-10-10 cuvette (10 mm path length), along with 3 mL of solvent transferred to a second cuvette to serve as a blank. The background-subtracted absorbance of the standard solution was measured to determine its concentration using reported values of the absorbance peaks and molar extinction coefficients of lycopene in the three solvents^1^ (Supplementary Table 4).

We used the lycopene standard to calibrate our Molecular Devices SpectraMax i3 plate reader. An absorbance spectrum showed that the three absorbance peaks for lycopene were at 449 nm, 475 nm, and 507 nm (Supplementary Fig. 19a). We prepared solutions ranging in concentration from 0 to 8 μg mL^−1^ from the main standard solution. We dispensed five 200 μL aliquots of each solution and a solvent blank into randomized wells on a polypropylene clear-bottom 96-well plate. We measured absorbance of the plate at 475 nm and 507 nm, and used these values to generate a calibration curve (Supplementary Fig. 19b). This calibration was performed twice, 4 months apart, and similar curves were obtained in both calibrations. We averaged the slope of the two curves for calibrating lycopene absorbance measurements.

To calibrate measurements of optical density at 700 nm (because RFP absorbs at 600 nm^2^) on the plate reader to dry cell mass, we inoculated 50 mL of TB in a 250 mL shake flask with a single colony of cells, and incubated it for 24 h. From this culture, we created six different 20 mL cultures of cells in 50 mL Falcon tubes with OD_700_ ranging from 1 to 6, using PBS for the dilutions. We removed 250 μL from each and diluted again with 1 mL of PBS. We added five 200 μL aliquots from each dilution and a PBS blank to a randomly assigned well on a clear-bottom polystyrene 96-well plate, and measured the OD_700_ for each well.

Falcon tubes with the 20 mL cultures were centrifuged at 6000 × *g* for 10 min. The supernatant was removed, the cells were resuspended in PBS, and washed. The cells were centrifuged again at 6000 × *g* for 10 min, the supernatant was poured off, and all remaining liquid aspirated. The tubes were placed uncovered in a drying oven at 105 °C for 24 h. The tubes were then removed, covered, and allowed to cool to room temperature. The dried cell pellets were carefully removed and weighed on a balance with 0.1 mg of precision. The absorbance measurements and cell pellet masses were used to generate a calibration curve (Supplementary Fig. 20). The calibration was performed twice, and we averaged the slopes of the two curves to calibrate OD_700_ to dry cell mass.

### Assay performance

At the conclusion of the experimental growth time, a 250 μL aliquot from each culture was assayed for dry cell mass and titer. The 250 μL aliquot (in a deep-well 96-well plate) was diluted with 1 mL of PBS. A 40 μL aliquot of the diluted cells was transferred into 160 μL of PBS in a clear-bottom polystyrene 96-well plate and mixed. Absorbance at 700 nm was measured on a Molecular Devices SpectraMax i3 plate reader. For cells expressing RFP, fluorescence was also measured on the plate reader with excitation at 585 nm and emission at 625 nm.

For cells expressing lycopene, which accumulates in the cytoplasmic membrane^32^, the remaining 1210 μL of the diluted cells were transferred to a 1.5 mL microcentrifuge tube, and centrifuged on an Eppendorf MiniSpin microcentrifuge with F-45-12-11 rotor at 1100×*g* (4000 rpm) for 4 min. The supernatant was aspirated, and then lycopene was extracted using a modified version of a previously published protocol^33^. In total, 250 μL of methanol was added, and the tubes were vortexed vigorously for 5 s to break up the cell pellet. A pipette tip was then used to further break up the pellet. In total, 250 μL of acetone was added, and the tubes were vortexed vigorously for 5 s again. Then, 250 μL of dichloromethane was added, the tubes were vortexed vigorously for 5 s, and then allowed to incubate at room temperature for 10 min to complete the extraction of lycopene from the cell pellets. The tubes were centrifuged at 12,000 ×*g* (13,400 rpm) for 5 min to pellet the cell debris, and then 200 μL aliquots were transferred to randomly assigned wells on a clear-bottom polypropylene 96-well plate, along with four aliquots of solvent blanks. During the aliquoting process, the plate was covered with a polypropylene plate cover to minimize evaporation, which is a concern when working with small volumes of volatile solvents. Absorbance at 475 nm, 507 nm, and 600 nm was measured in the plate reader. Lycopene does not absorb at 600 nm. Absorbance at 600 nm was used to detect the presence of contamination in the samples. The calibration curves were applied to measurements of absorbance at 475 nm and 507 nm to estimate lycopene concentration at these two absorbance peaks, and then averaged to determine the lycopene concentration in the sample.

### Data analysis

All raw and processed data are available online (Supplementary Data 4), at doi: 10.6084/m9.figshare.6848957, and https://github.com/arielhecht/cell-metrics. Absorbance and fluorescence measurements were collected through the Molecular Devices SoftMax Pro 6.4 software. All data analysis was performed in R with the FrF2^34^ package for fractional factorial design and analysis, tidyverse^35^ packages for data transformation, and the ggplot2^36^ package for figure generation.

Repeatability is the closeness of the agreement between the results of successive measurements of the same measurand carried out under the same conditions of measurement^37^. Reproducibility is the closeness of the agreement between the results of measurements of the same measurand carried out under changed conditions of measurements (which may include principle of measurement, method of measurement, observer, measuring instrument, reference standard, location, conditions of use, or time)^37^. We evaluated reproducibility under changed conditions of use and time.

In this paper, we define titer as the amount of product produced (mg of lycopene or arbitrary fluorescence units of RFP) per volume of culture. We define dry cell mass as the mass of dry cells per volume of culture. We define yield as the ratio of the amount of product produced per mass of dry cells, which is the quotient of titer over yield.

Relative effect magnitude is the absolute value of the difference between the mean response at each level divided by the overall mean response. For one factor, *X*_*i*_, at two levels, − and + , with one response *Y*, the relative effect magnitude is

3 $${\mathrm{REM}}_{X_i} = \left| {\frac{{\bar Y_{X_{i + }} - \bar Y_{X_{i - }}}}{{\bar Y}}} \right|$$

For two factors, *X*_*i*_ and *X*_*j*_, the relative effect magnitude is

4 $${\mathrm{REM}}_{X_iX_j} = \left| {\frac{{\bar Y_{X_{i + }X_{j + }} + \bar Y_{X_{i - }X_{j - }} - \bar Y_{X_{i + }X_{j - }} - \bar Y_{X_{i - }X_{j + }}}}{{\bar Y}}} \right|$$

### Code availability

All R scripts used to generate the experimental designs and analyze the experimental data are available online at https://github.com/arielhecht/cell-metrics.

## Electronic supplementary material

Supplementary Information

Supplementary Data 1

Supplementary Data 2

Supplementary Data 3

Supplementary Data 4

## Data Availability

Source data for Fig. 1 and Supplementary Figs. 2–16 have been provided in Supplementary Data 4. All data are available online at 10.6084/m9.figshare.6848957.v1^38^. All other data supporting the findings of this study are available from the corresponding author on request.
