# Supplementary material for: A minimum information standard for reproducing bench-scale bacterial cell growth and productivity
Source: Commun Biol. 2018 Dec 6;1:219. doi: 10.1038/s42003-018-0220-6 (PMC6283831; doi:10.1038/s42003-018-0220-6)
Supplement: Supplementary file 1 — Supplementary Information [file 42003_2018_220_MOESM1_ESM.pdf]

Supplementary Figures

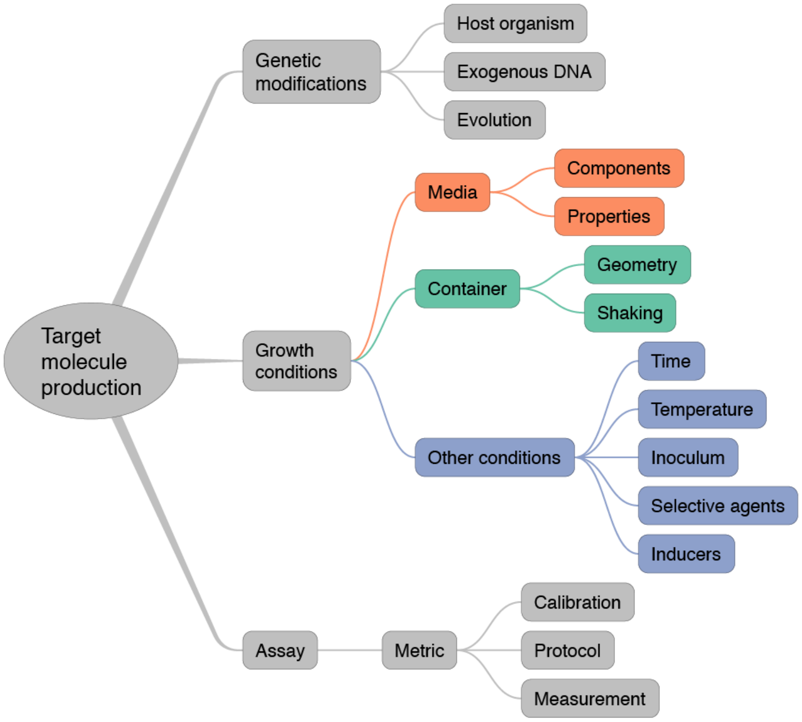

Supplementary Figure 1--The process of engineering an organism towards a specific objective, such as producing a target molecule, proceeds in three steps: genetically modifying the organism, growing the organism, and assaying its function. The focus of this paper is on growth conditions. Growth condition factors can be broken down into three broad categories: media, container, and other conditions such as time and temperature.

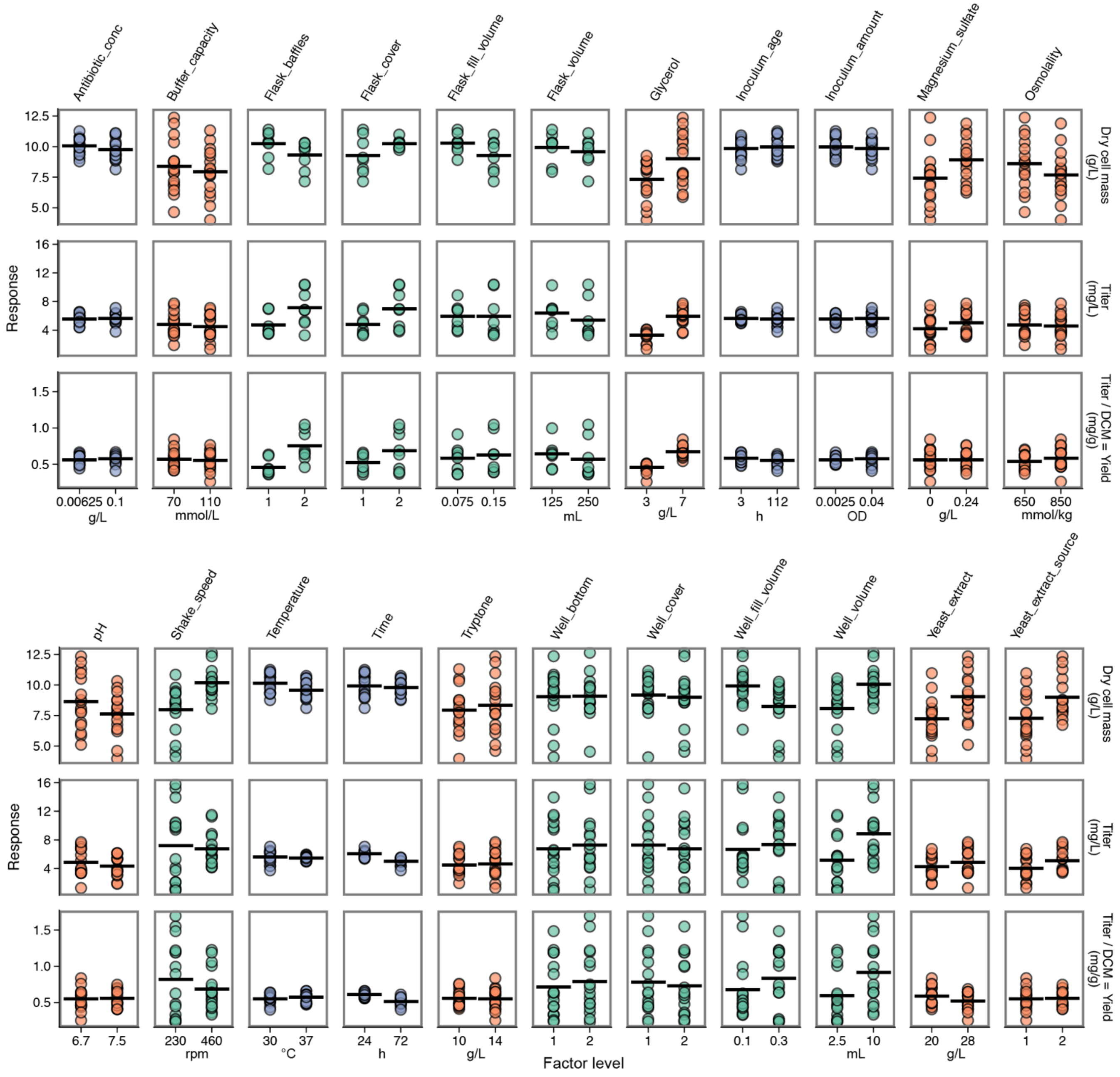

Supplementary Figure 2--Main effects plot for lycopene-producing strains. Data from Group 1 experiments, and from the experimental sub-groups in which the factor is varied. Fill color corresponds to the factor category. Horizontal lines represent the mean response at each factor level. For flask baffles, 1 = unbaffled, 2 = baffled. For flask cover, 1 = foam, 2 = aluminum foil. For well bottom, 1 = pyramidal, 2 = round. For well cover, 1 = aeraseal membrane, 2 = aluminum foil. For yeast extract source, 1 = Sigma, 2 = Millipore.

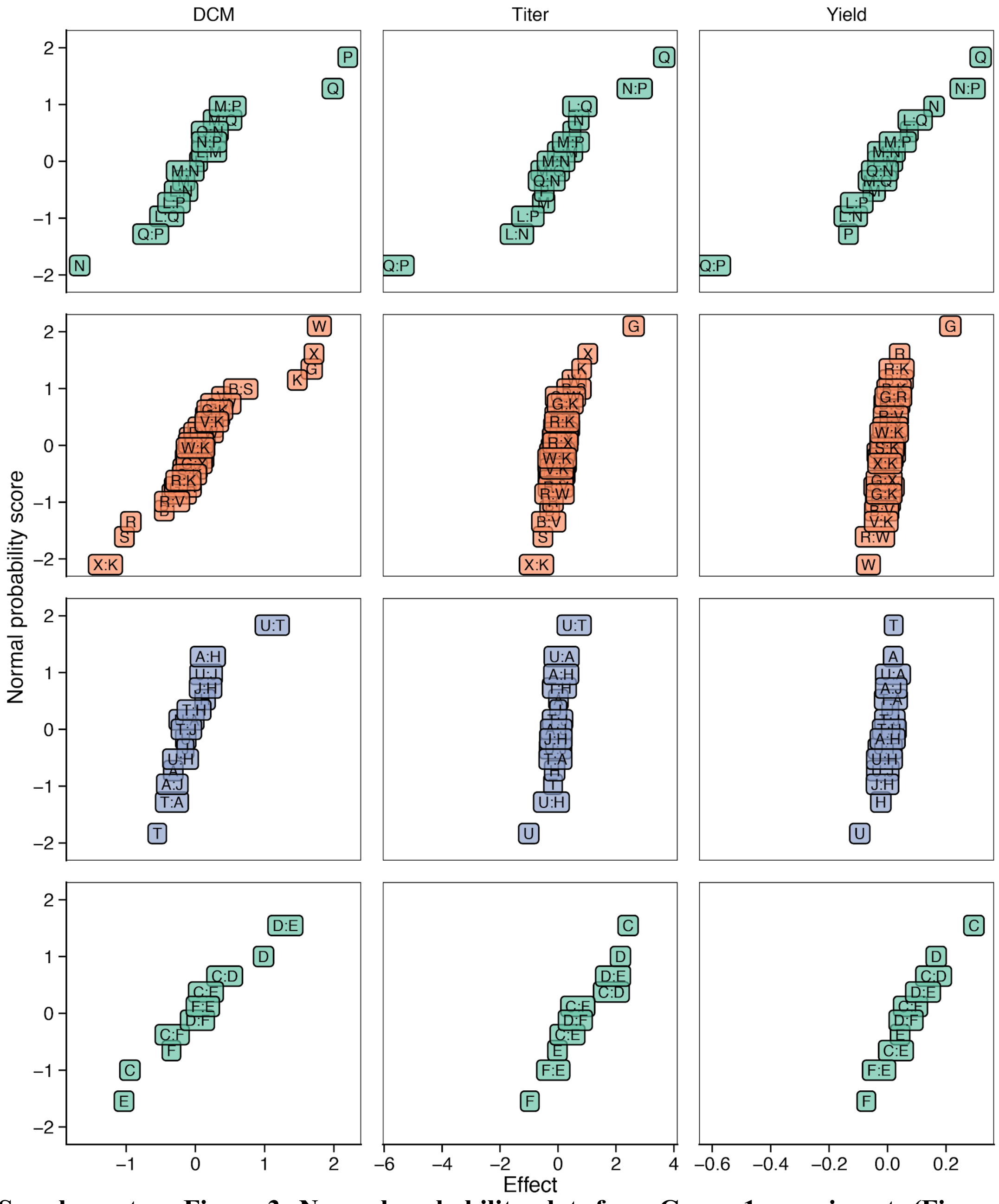

Letter	Factor
C	Flask baffles
D	Flask cover
E	Flask fill volume
F	Flask volume
L	Well bottom
M	Well cover
N	Well fill volume
P	Shake speed
Q	Well volume
B	Buffer capacity
G	Glycerol
K	Magnesium sulfate
R	Osmolality
S	pH
V	Tryptone
W	Yeast extract
X	Yeast extract source
A	Antibiotic concentration
H	Inoculum age
J	Inoculum amount
T	Temperature
U	Time

Supplementary Figure 3--Normal probability plots from Group 1 experiments (Figure 1a). Horizontal axis is the effect of the factor, or the two-factor interaction. Vertical axis is the normal probability score. Factors that deviate from the straight line that fits the bulk of the factors have an effect greater than would be expected from a normal distribution, and have significant effects. The significant two-factor interactions are Q:P, N:P, X:K, U:T, D:E.

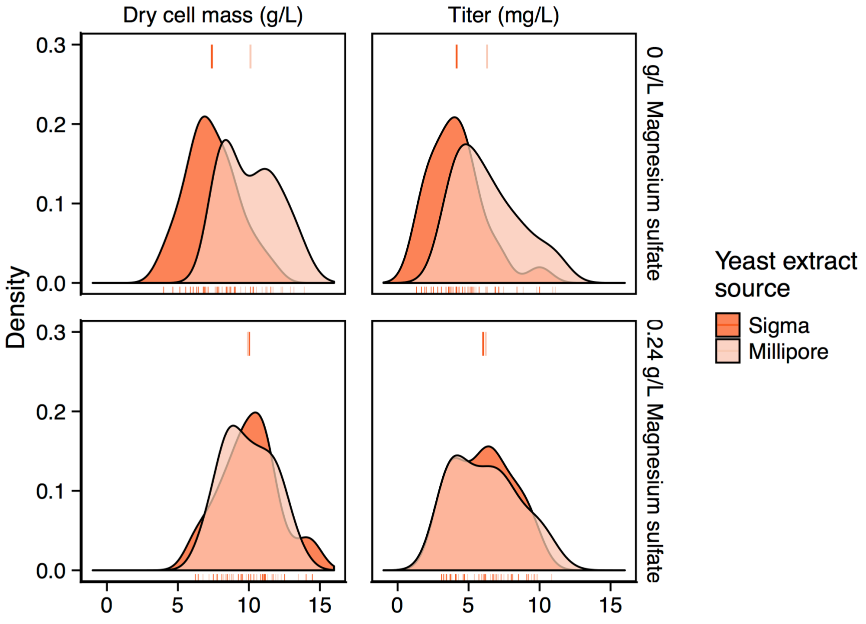

Supplementary Figure 4--Two-factor interactions between yeast extract source and magnesium sulfate concentration for lycopene production. Yeast extract, a common ingredient in bacterial growth media and the primary source of most nutrients in our media, does not have a defined composition or preparation protocol. Recipes for media containing yeast extract typically do not recommend supplementing magnesium, despite the fact that magnesium ions are an essential nutrient,[5] and different preparations of yeast extract can be deficient in magnesium.[6] It was previously reported that the addition of 2 mM (0.24 g L^{-1}) magnesium sulfate to media can eliminate the difference between different sources of yeast extract.[6] We observed a 37% difference in dry cell mass and a 52% difference in lycopene titer from cells grown with yeast extract from two different commercial sources in the absence of supplemental magnesium sulfate. This difference disappeared upon the addition of 0.24 g L^{-1} magnesium sulfate to both yeast extract sources, exactly as previously reported.[6] This results shows that media containing yeast extract should be supplemented with 0.24 g L^{-1} magnesium sulfate to improve reproducibility. Vertical ticks at the top of each panel represent the mean of each population.

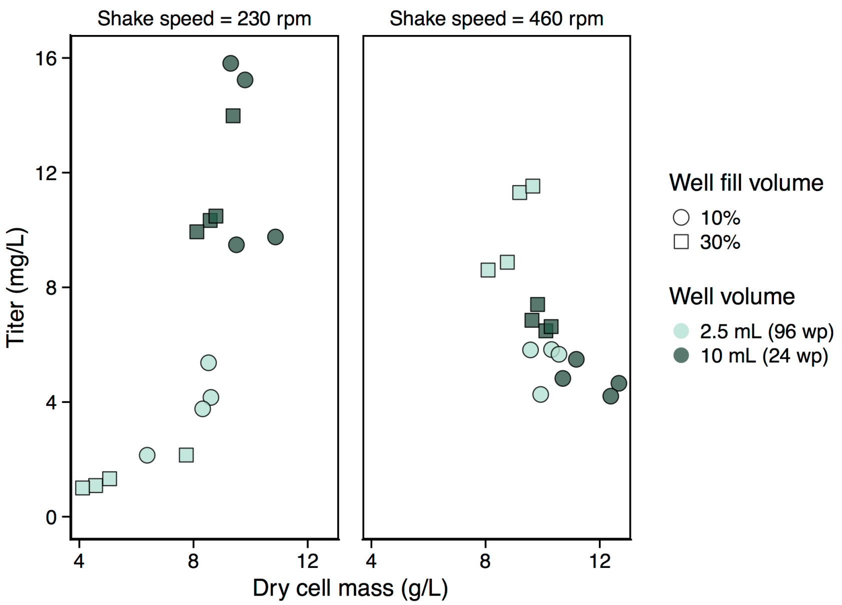

Supplementary Figure 5--Two-factor interactions between the microwell container factors shake speed, well volume and well fill volume were non-linear for lycopene production. Shake speed has a strong interaction with well volume and well fill volume. At low shake speed, titer is strongly positively affected by well volume, with minimal effect from well fill volume. At high shake speed, titer is moderately positively affected by well fill volume, and slightly negatively affected by well volume. This result highlights the need to exercise caution with container geometry factors, because differences in factor levels can lead to unpredictable results. A different set of non-linear interactions were observed between flask baffles, flask volume and flask fill volume (**Supplementary Fig. 7**).

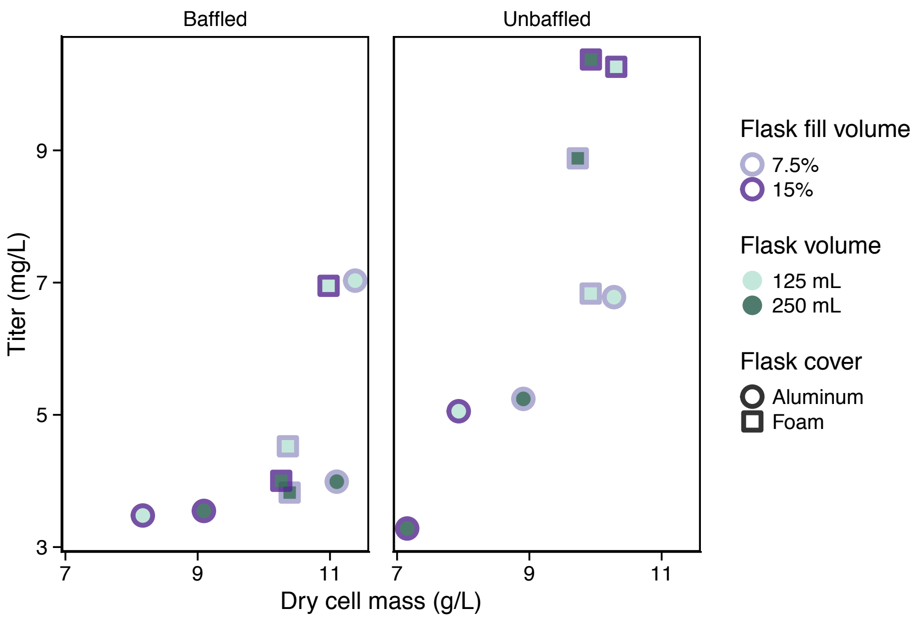

Supplementary Figure 6--Two-factor interactions between flask geometry factors for lycopene production. Point edge color represents flask fill volume, point fill color represent flask volume, and point shape represents flask cover. Lycopene titer is higher in unbaffled flasks than in baffled flasks. In baffled flasks, flask volume has the largest effect on titer and flask fill volume has the largest effect on dry cell mass. In unbaffled flasks, flask cover has the largest effect on titer and dry cell mass.

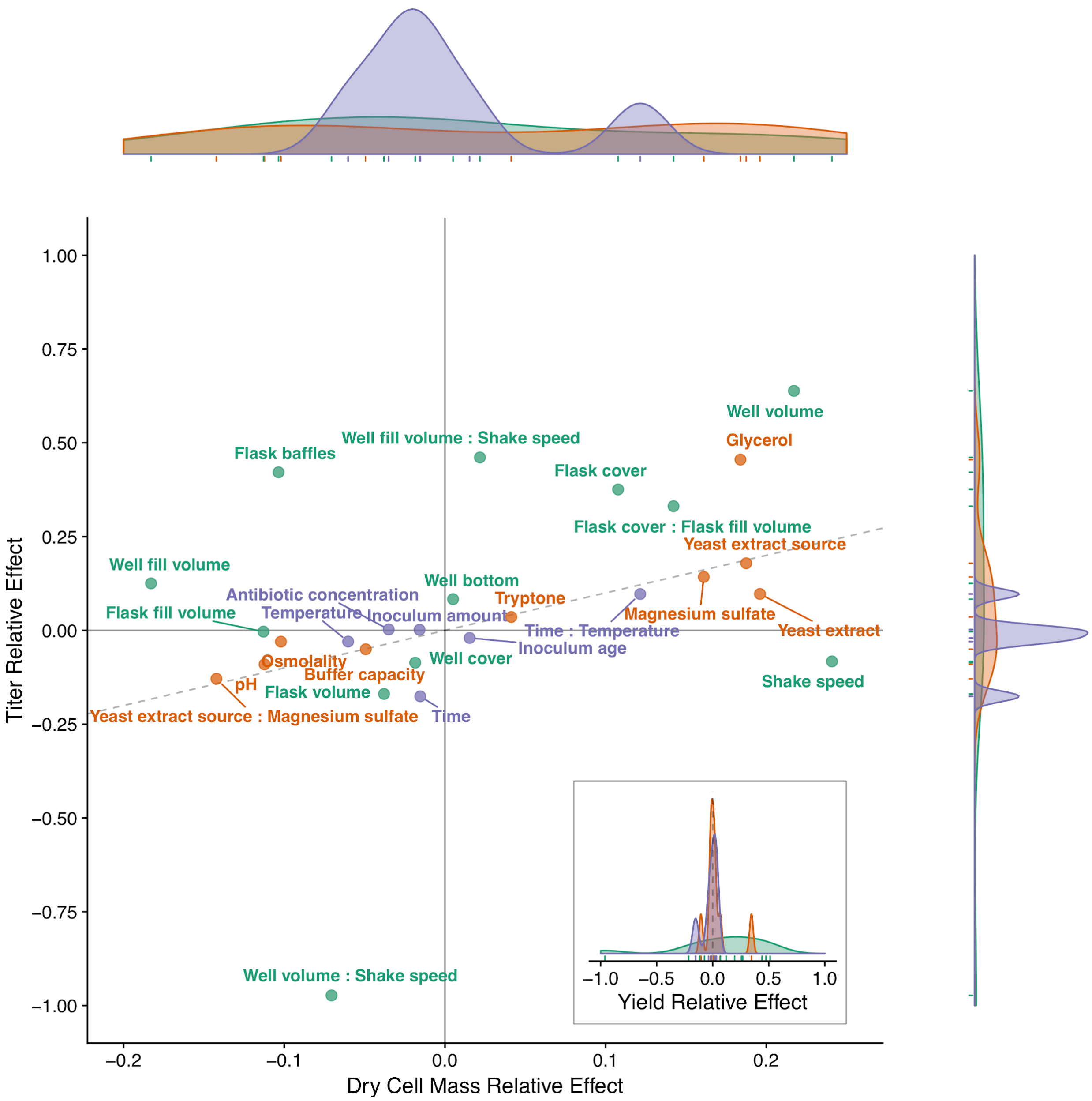

Supplementary Figure 7--Scatter plot of titer and dry cell mass relative effects for lycopene production. The relative effect of each factor and two-factor interaction **(Figure 1c)** is plotted, with directionality of the effect preserved. Dry cell mass relative effects are on the horizontal axis, and titer relative effects are on the vertical axis. The dashed grey line is the line of equal effect on titer and dry cell mass, which corresponds to no effect on yield. Density plots of factor effects by factor category for dry cell mass and titer are on the outside of the plot (yield is in the inset).

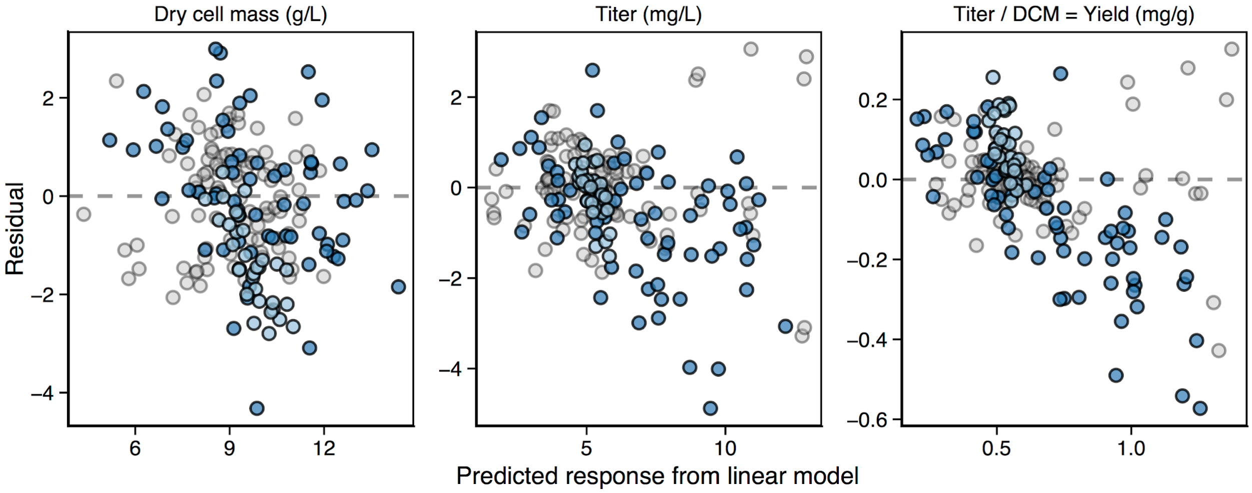

Supplementary Figure 8--Residual plot of linear model (Figure 1e). Light grey points are the model training data (Group 1). Dark blue and light blue points are the two sub-groups of the model test data (Group 2). The residuals are randomly distributed, with no significant structure, except for yield, which shows a negative trend in the residuals of the test data.

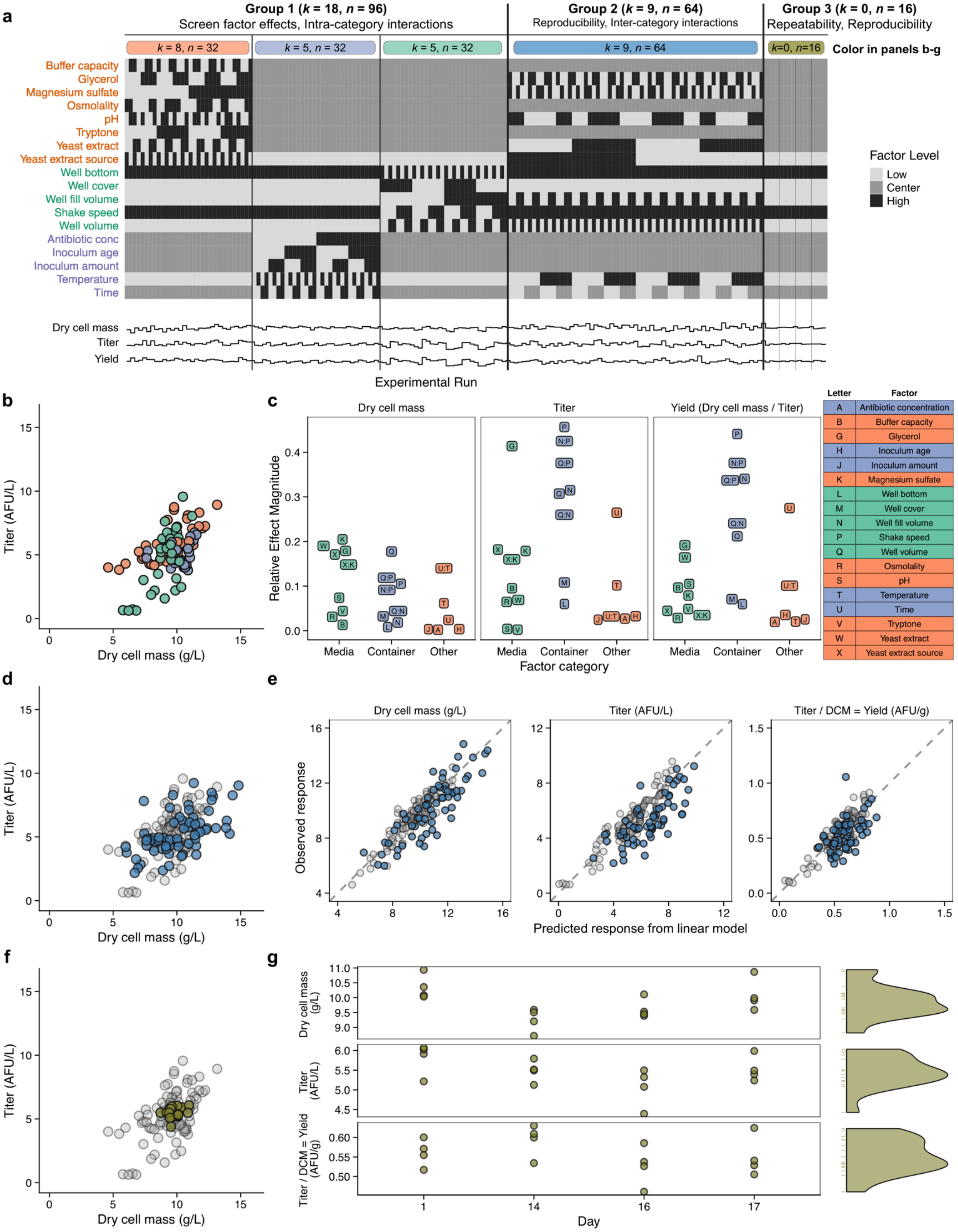

a
Group 1 (k = 18, n = 96)
Screen factor effects, Intra-category interactions
Group 2 (k = 9, n = 64)
Reproducibility, Inter-category interactions
Group 3 (k = 0, n = 16)
Repeatability, Reproducibility
k = 8, n = 32
k = 5, n = 32
k = 5, n = 32
k = 9, n = 64
k=0, n=16
Color in panels b-g
Buffer capacity
Glycerol
Magnesium sulfate
Osmolality
pH
Tryptone
Yeast extract
Yeast extract source
Well bottom
Well cover
Well fill volume
Shake speed
Well volume
Antibiotic conc
Inoculum age
Inoculum amount
Temperature
Time
Factor Level
Low
Center
High
Dry cell mass
Titer
Yield
Experimental Run
b
Titer (AFU/L)
Dry cell mass (g/L)
c
Dry cell mass
Titer
Yield (Dry cell mass / Titer)
Relative Effect Magnitude
Media
Container
Other
Factor category
Letter
Factor
A Antibiotic concentration
B Buffer capacity
G Glycerol
H Inoculum age
J Inoculum amount
K Magnesium sulfate
L Well bottom
M Well cover
N Well fill volume
P Shake speed
Q Well volume
R Osmolality
S pH
T Temperature
U Time
V Tryptone
W Yeast extract
X Yeast extract source
d
Titer (AFU/L)
Dry cell mass (g/L)
e
Dry cell mass (g/L)
Titer (AFU/L)
Titer / DCM = Yield (AFU/g)
Observed response
Predicted response from linear model
f
Titer (AFU/L)
Dry cell mass (g/L)
g
Dry cell mass (g/L)
Titer (AFU/L)
Titer / DCM = Yield (AFU/g)
Day

Supplementary Figure 9--Repeatability and reproducibility of factor effects on cell growth and productivity for RFP. (a) Experimental design table with 18 factor rows, and 176 experimental run columns. Colored bars at the top correspond to color of points in other panels. *k* is the number of factors varied, and *n* is the number of runs, in a group or experiment. The three responses, normalized to range from 0 to 1, are below. (b) Dynamic range of lycopene titer and dry cell mass observed in Group 1 experiments. (c) Relative effect magnitude of all 18 factors on all 3 responses, colored by factor category. (d) Dynamic range of titer and dry cell mass observed in Group 2 experiments. (e) Factor effects observed in Group 1 are reproducible in Group 2. Grey points are Group 1 data used to train linear model (dry cell mass $r^2 = 0.85$, titer $r^2 = 0.89$, yield $r^2 = 0.82$). Blue points are Group 2 data used to test linear model (dry cell mass $r^2 = 0.74$, titer $r^2 = 0.59$, yield $r^2 = 0.27$). (f) Dynamic range of titer and dry cell mass observed in Group 3 centerpoint replicates. (g) Centerpoint replicates plotted as a function of day on which they were run show no trends over time.

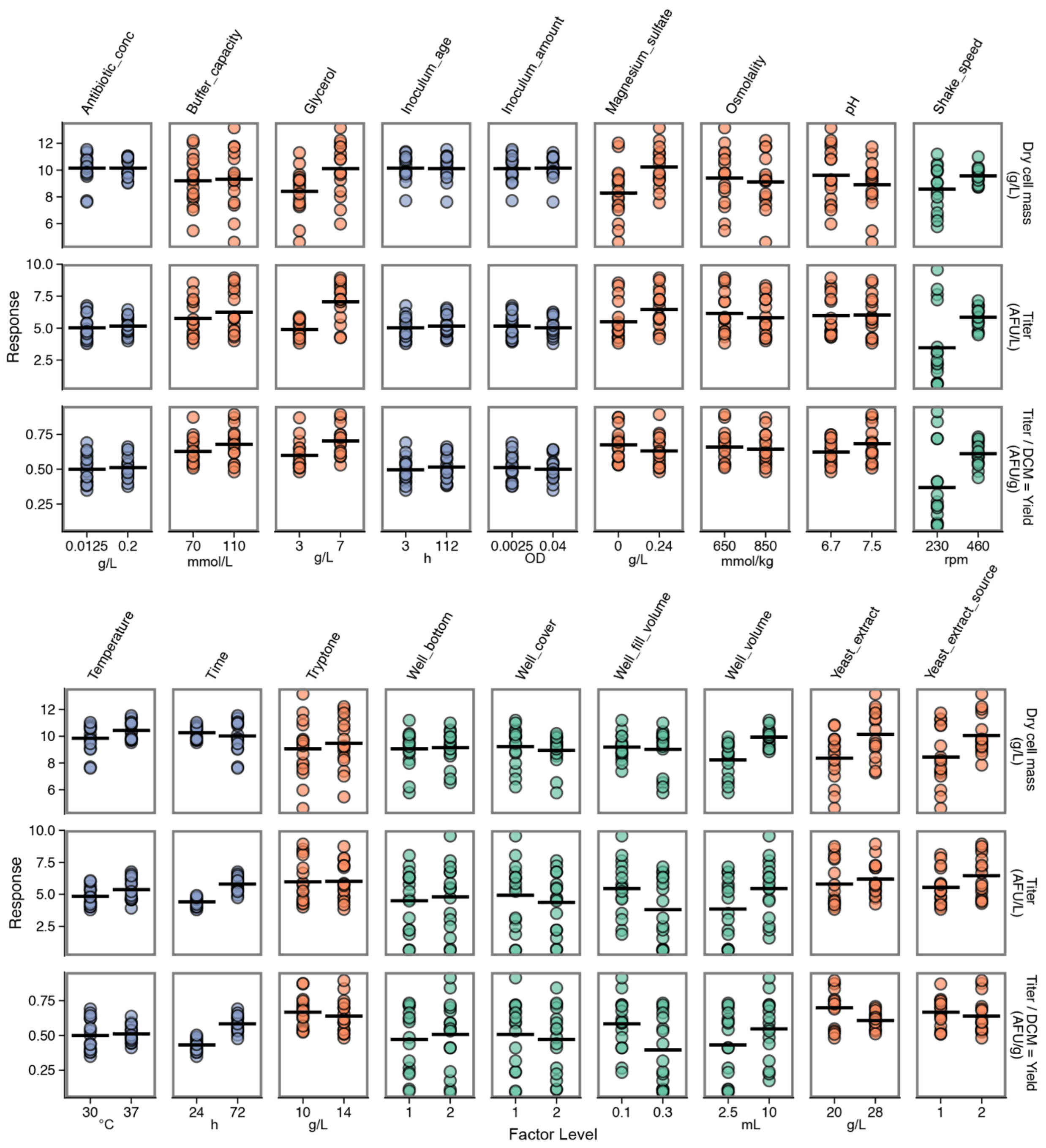

Supplementary Figure 10--Main effects plot for RFP-producing strains. Data from Group 1 experiments, and from the experimental sub-groups in which the factor is varied. Fill color corresponds to the factor category. Horizontal lines represent the mean response at each factor level. For well bottom, 1 = pyramidal, 2 = round. For well cover, 1 = aeraseal membrane, 2 = aluminum foil. For yeast extract source, 1 = Sigma, 2 = Millipore.

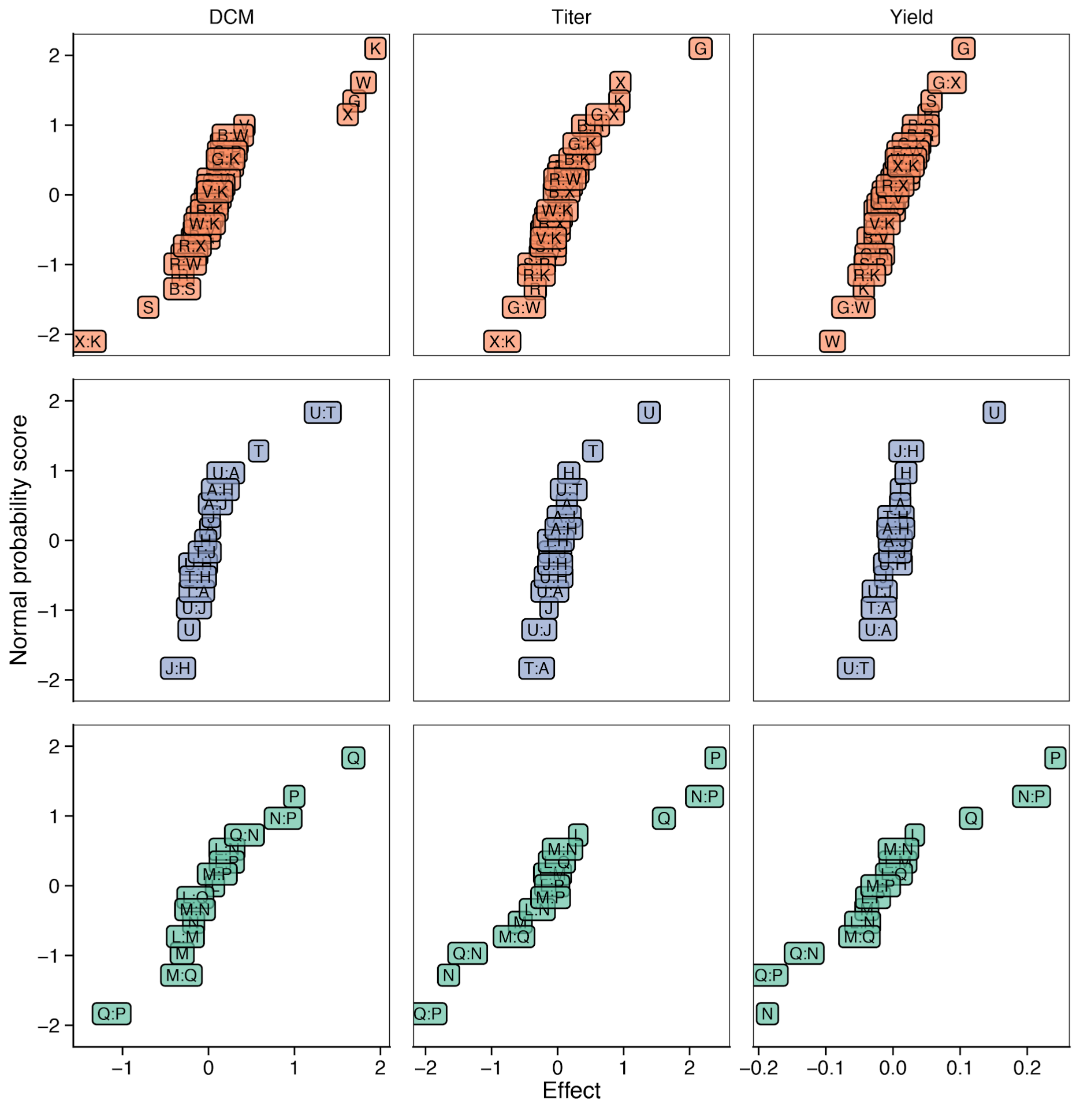

Letter	Factor
B	Buffer capacity
G	Glycerol
K	Magnesium sulfate
R	Osmolality
S	pH
V	Tryptone
W	Yeast extract
X	Yeast extract source
A	Antibiotic concentration
H	Inoculum age
J	Inoculum amount
T	Temperature
U	Time
L	Well bottom
M	Well cover
N	Well fill volume
P	Shake speed
Q	Well volume

Supplementary Figure 11--Normal probability plots from Group 1 experiments (Supplementary Fig. 8a), for all 3 responses. Horizontal axis is the effect of the factor, or the two-factor interaction. Vertical axis is the normal probability score. Factors that deviate from the straight line that fits the bulk of the factors have an effect greater than would be expected from a normal distribution, and have significant effects. The significant two-factor interactions are X:K, U:T, Q:P, N:P, Q:N.

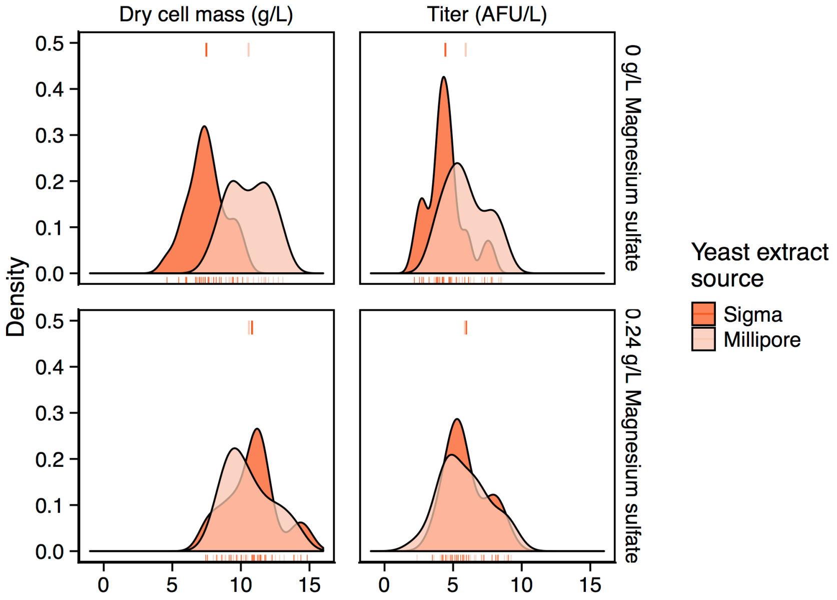

Supplementary Figure 12--Two-factor interactions between yeast extract source and magnesium sulfate concentration for RFP production. Supplemental magnesium sulfate can make up for magnesium deficiency in different sources of yeast extract. Vertical ticks at the top of each panel represent the mean of each population. Without supplemental magnesium sulfate, two different sources of yeast extract result in a 40% difference in dry cell mass and 34% difference in titer. With 0.24 g L^{-1} magnesium sulfate added to the media, there is no difference in either response between the two sources of yeast extract.

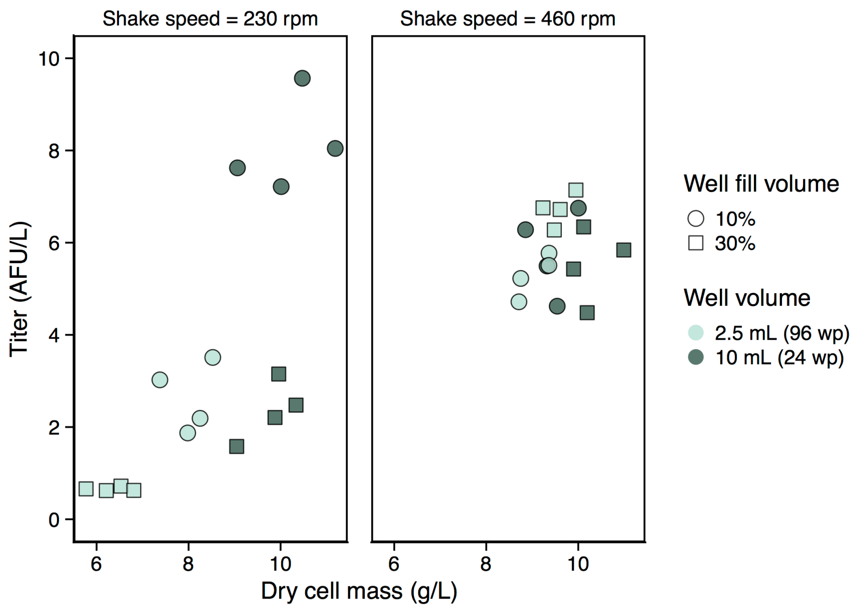

Supplementary Figure 13--Two-factor interactions between the microwell container factors shake speed, well volume and well fill volume were non-linear for lycopene production. At low shake speeds, well volume and well fill volume both have a large effect on titer and dry cell mass. At high shake speeds, the effects of well volume and well fill volume are diminished.

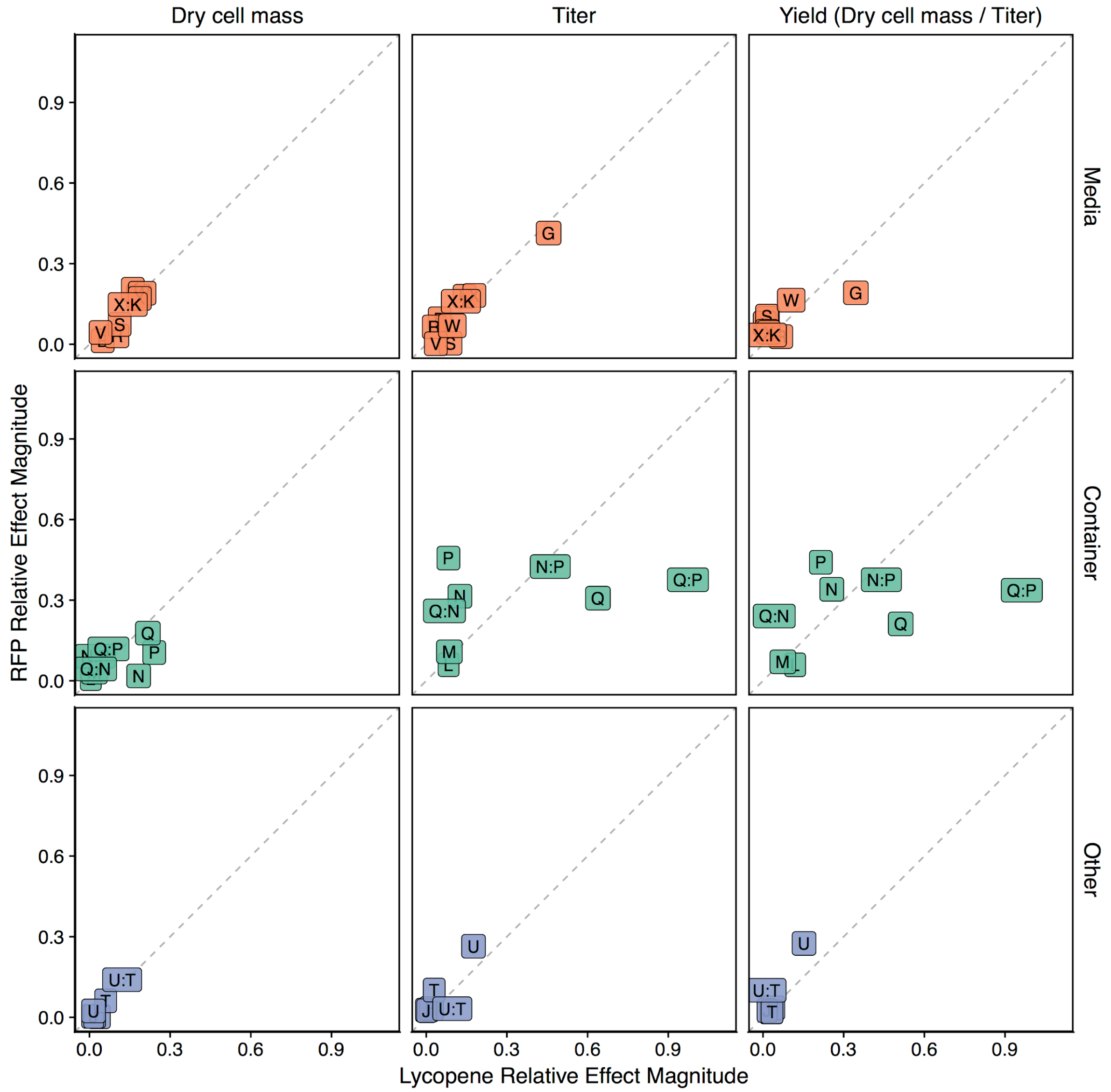

Supplementary Figure 14--Comparison of relative effect magnitude of factors and two-factor interactions for cells engineered to produce lycopene (horizontal axis) and RFP (vertical axis). Plots are facetted by response and factor category. Factors and interactions have similar effects on both types of cells except for the container factors on titer and yield, which show different effects for cells producing lycopene and RFP. This may be due to the different biosynthetic routes the two products take -- lycopene is a small molecule derived from central metabolism, and RFP is a heterologous protein.

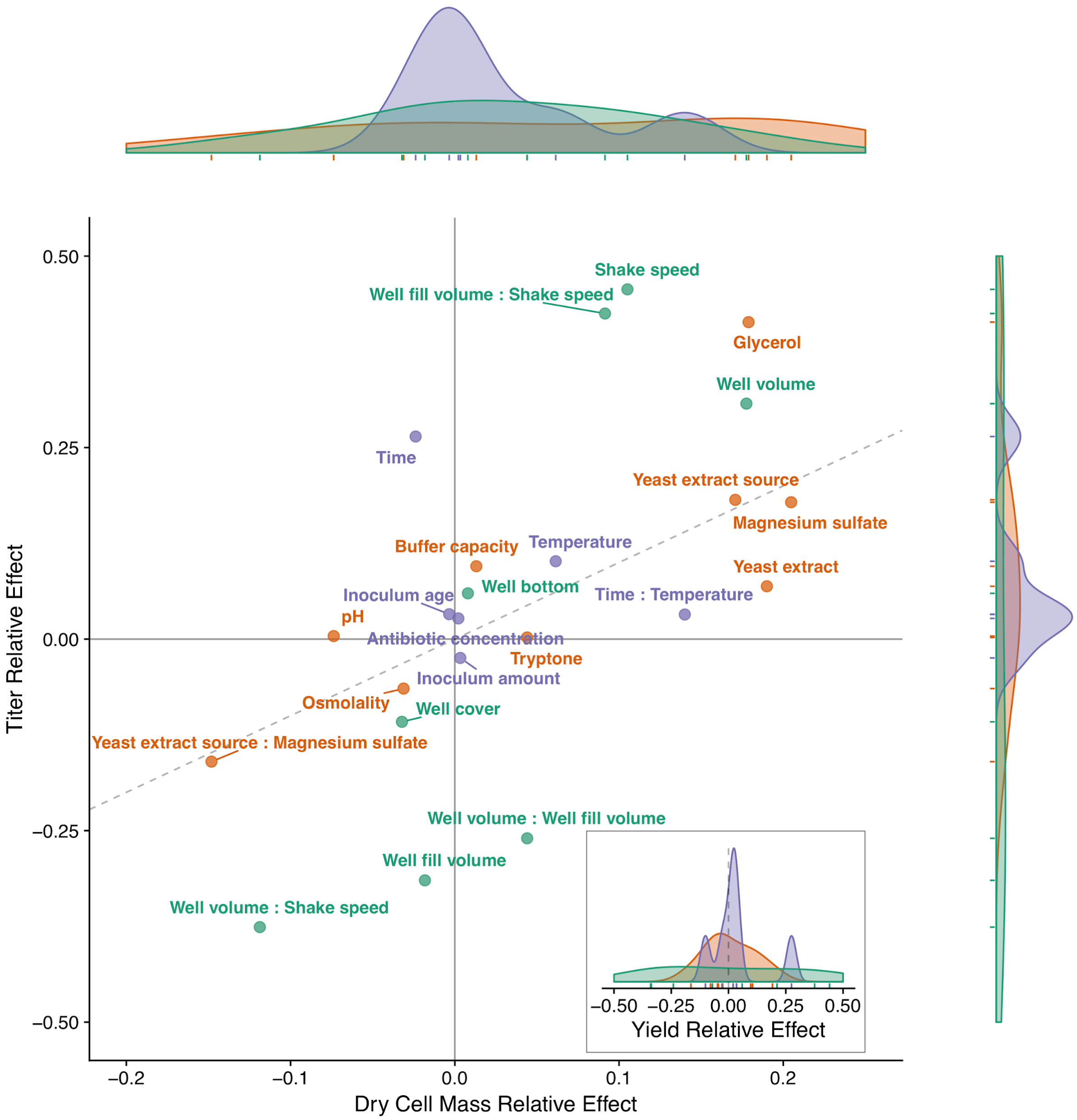

Supplementary Figure 15--Scatter plot of titer and dry cell mass relative effects for RFP production. The relative effect of each factor and two-factor interaction **(Supplementary Fig. 9c)** is plotted, with directionality of the effect preserved. Dry cell mass relative effects are on the horizontal axis, and titer relative effects are on the vertical axis. The dashed grey line is the line of equal effect on titer and dry cell mass, which corresponds to no effect on yield. Density plots of factor effects by factor category for dry cell mass and titer are on the outside of the plot (yield is in the inset).

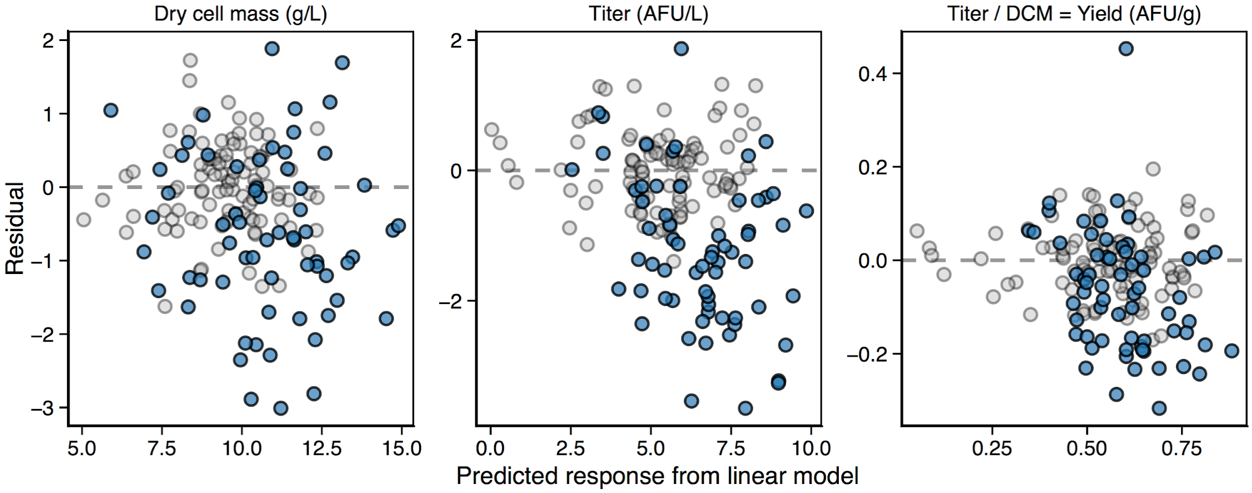

Supplementary Figure 16--Residual plot of linear model (Supplementary Fig. 8e). Light grey points are the model training data (Group 1). Dark blue points are the two sub-groups of the model test data (Group 2). The residuals are randomly distributed, with no significant structure, except for the titer residuals, which have a negative bias.

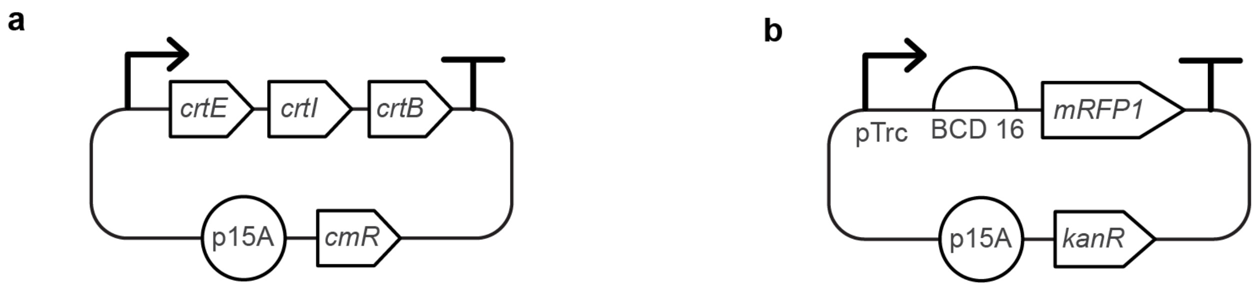

Supplementary Figure 17--Plasmid maps. (a) Map of the pAC-LYC plasmid. The crt*EIB* operon is under the control of a single constitutive promoter, on a low-copy p15A plasmid with the chloramphenicol resistance gene. (b) Map of pFAB3992 plasmid. The mRFP1 gene is under the control of the constitutive pTrc promoter, with translation initiation through a bicistronic design RBS, on a low-copy p15A plasmid with the kanamycin resistance gene.

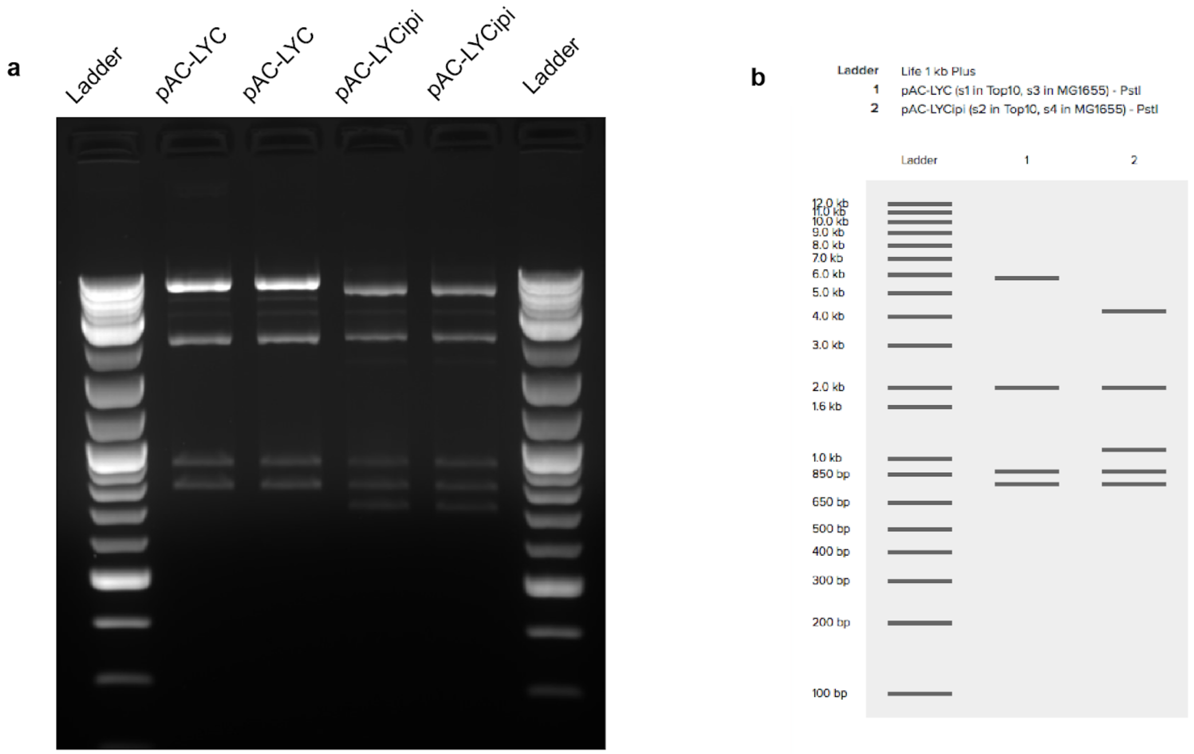

Supplementary Figure 18--Plasmid digestion by PstI to confirm identity. We initially received two lycopene producing plasmids from Addgene, pAC-LYC, which we used in this paper, and pAC-LYCipi (Addgene plasmid #53279). pAC-LYCipi has an additional gene (idi) that should increase flux to the lycopene pathway.[7] Preliminary measurements indicated that lycopene titer was higher in strains transformed with pAC-LYC than with pAC-LYCipi. (a) We digested both plasmids with PstI in duplicate, and (b) expected to see 4 bands for pAC-LYC and 5 bands for pAC-LYCipi. The 4 pAC-LYC bands all appear to be the correct size. 4 of the 5 pAC-LYCipi bands appear to be the correct size, but one appears to be incorrect. We expected to see a 1.1 kb band, which instead was around 0.5 kb. This band mapped to the region of the plasmid carrying the idi gene, leading us to conclude that there was likely a part of this gene missing, which was affecting the lycopene titer from cells transformed with this plasmid. The pAC-LYC plasmid which we used for transforming our cells has the correct fragment sizes.

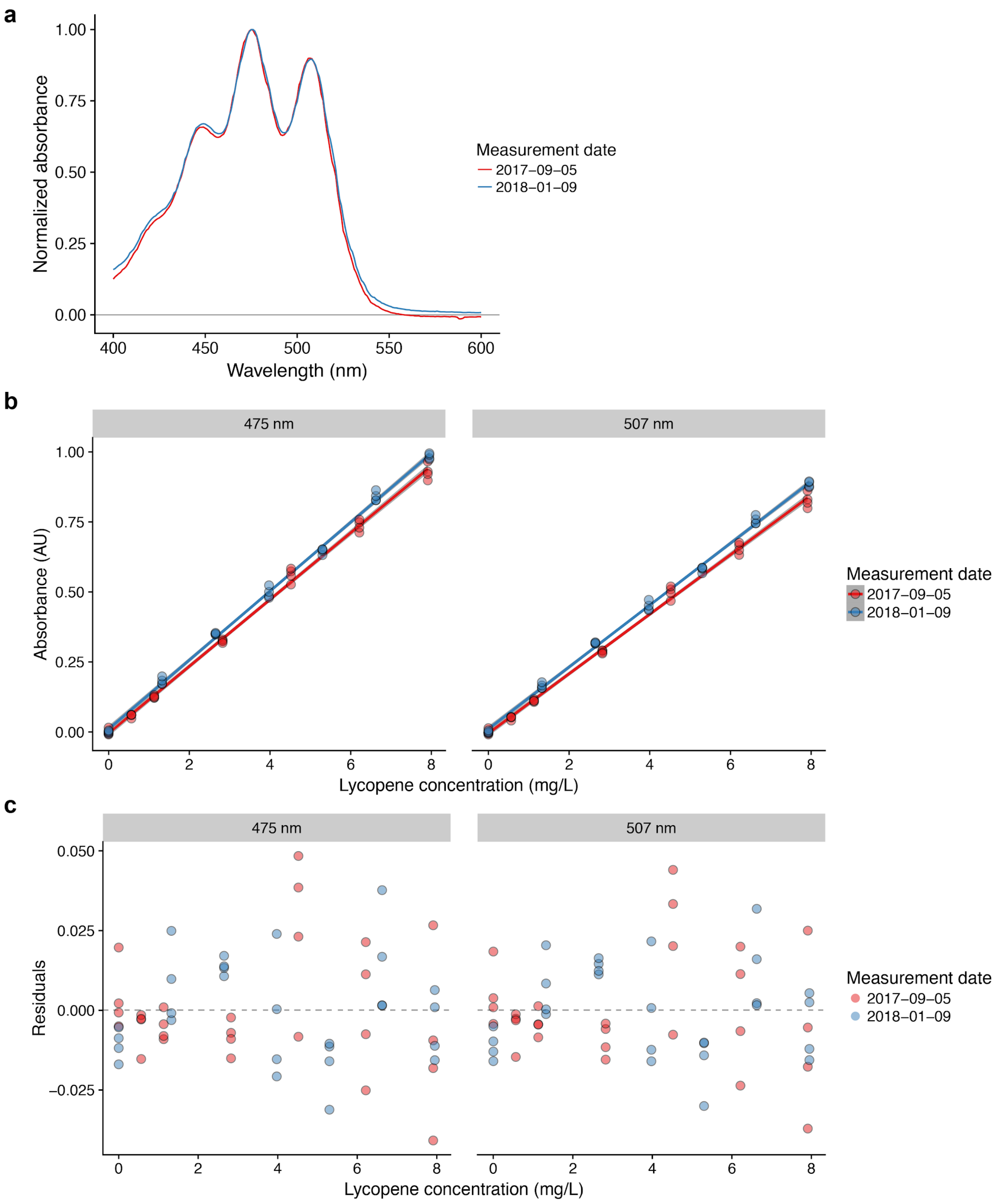

Supplementary Figure 19--Calibration curves for lycopene standard. (a) Normalized absorbance spectrum of lycopene standard measured on two different days, with peaks at 449 nm, 475 nm and 507 nm. (b) Calibration curves of lycopene concentration (horizontal axis) against measured absorbance (vertical axis) as obtained on two different days from two different standard lots measured at 475 nm and 507 nm. Four aliquots from each standard concentration were measured from randomly-assigned wells. (c) Plot of the residuals shows that absorbance is linearly proportional to lycopene concentration over the

range of 0-8 mg L^{-1}. For calibration of lycopene measurements, we calculated a calibration curve using both sets of calibration points. For absorbance at 475 nm, our calibration curve has a slope of 0.122 with an intercept of 0.002 (r^2 = 0.995). For absorbance at 507 nm, our calibration curve has a slope of 0.109 with an intercept of 0.002 (r^2 = 0.994).

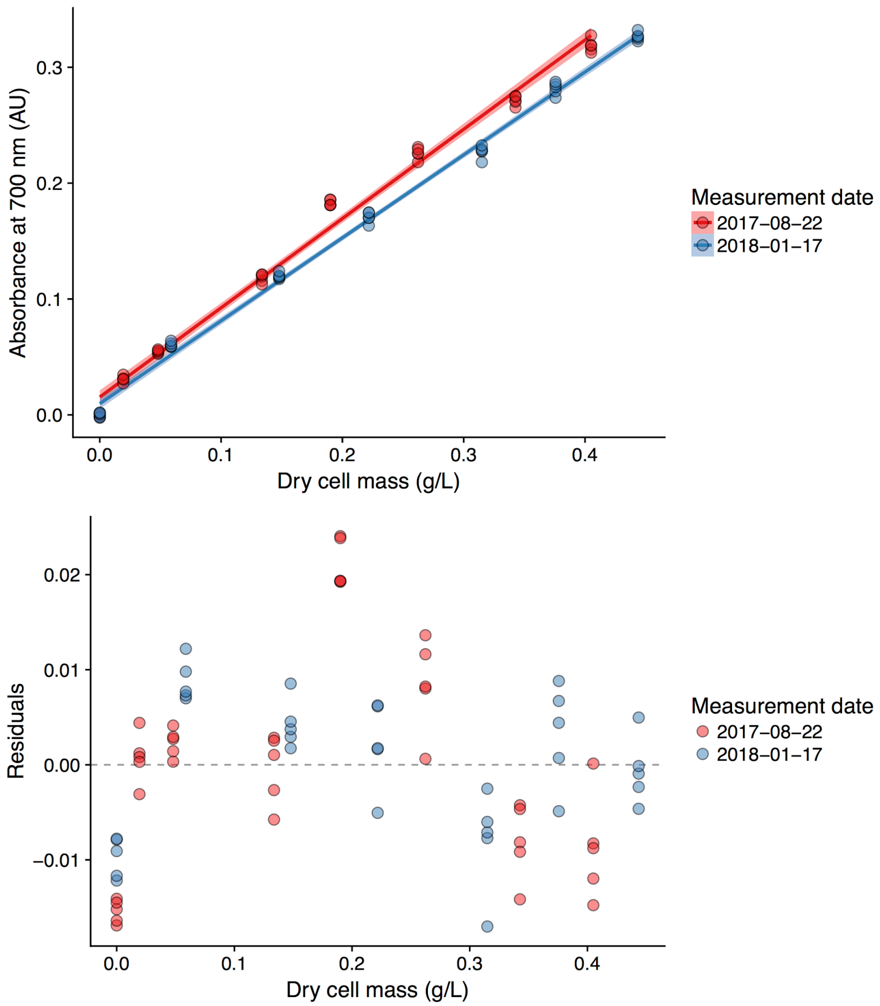

Supplementary Figure 20--Calibration curves for dry cell mass. (top) Calibration curves of absorbance measured at 700 nm (vertical axis) as a function of dry cell mass (horizontal axis). The calibration was performed on two different days. Absorbance of five aliquots from each concentration were measured from randomly-assigned wells. (bottom) Plot of the residuals shows that absorbance is approximately linear to dry cell mass over the range of 0-0.45 g L^{-1}, although slight curvature in the residuals is visible, indicating that we are approaching the top of the linear range of our plate reader. We calculated a calibration curve using both sets of calibration points. Our calibration curve has a slope of 1.34 with an intercept of -0.016 (r^2 = 0.986).

Supplementary Tables

Supplementary Table 1--Assessment of the homogeneity of the variance between replicates performed on different days, as measured by Levene's test,[4] for lycopene (Figure 1g) and RFP (Supplementary Figure S9g). p-values represent the likelihood of incorrect rejection of the null hypothesis of equal variance between the different days. These results indicate that we cannot reject the null hypothesis of equal variance, and that it is likely that the samples on different days are drawn from the same distribution.

Homogeneous variance (Levene's test)	**p-value, Dry cell mass**	**p-value, Titer**	**p-value, Yield**
Lycopene	0.994	0.647	0.545
RFP	0.934	0.858	0.969

Supplementary Table 2--List of reagents, manufacturers, catalog and lot numbers used in these experiments.

Reagent	Manufacturer	Catalog number	Lot number
Tryptone	Sigma-Aldrich	T7293-250G	SLBT3436
Yeast extract	Sigma-Aldrich	Y1625-250G	SLBR9838V
Yeast extract	Millipore	1.03753.0500	VM781753704
Sodium chloride	Sigma-Aldrich	S7653-250G	SLBQ5226V
Glycerol	Sigma-Aldrich	G5516-1L	SHBH2896
Potassium phosphate	Sigma-Aldrich	P5655-100G	SLBS0199V
Dipotassium phosphate	Sigma-Aldrich	P8584-1L	SLBR3106V
Magnesium sulfate	Sigma-Aldrich	83266-100ML-F	BCBQ7529V
Phosphate buffered saline	Sigma-Aldrich	D8537	RNBG1595
Deionized water (18 MΩ-cm)	Millipore	Milli-Q Advantage A10	(none)
Chloramphenicol	Sigma-Aldrich	C0378-5G	SLBW4488
Kanamycin	Sigma-Aldrich	K1377-5G	SLBR6873V
Calcium chloride	Sigma-Aldrich	793639-100G	MKCD2331
SOC media	New England Biolabs	B9020S	4841612
Bacto agar	BD	214010	3220297
QIAprep Spin Miniprep Kit	Qiagen	27104	148045596
1.5 mL LoBind microcentrifuge tubes	Eppendorf	022431021	F169778P
PstI-HF	New England Biolabs	R3140S	0011308
96-well plate, polystyrene, clear-bottom	Grenier	655090	E17013BQ
96-well plate, polypropylene, clear-bottom	Grenier	655201	E16093KU
Polypropylene plate cover	Thermo Scientific	AB-0755	(none)
96-well plate, round bottom	Ritter	43001-0020	(none)
96-well plate, pyramidal bottom	Corning Life Science	3960	02017000
24-well plate, round bottom	Whatman	WHA77015102	A000292306

24-well plate, pyramidal bottom	E&K Scientific	EK-2053-S	02317424
125 mL baffled flask	Corning	4444-125	(none)
125 mL unbaffled flask	Corning	4442-125	04117036
250 mL baffled flask	Corning	4444-250	(none)
250 mL unbaffled flask	Corning	4442-250	29516001
AeraSeal Adhesive Microplate Seals	E&K Scientific	T896100	BD015A
Microseal 'F' foil seals	Bio-Rad	MSF1001	BR00439191
25 mm silicone foam closure	Bellco Glass	2004-00003	(none)
38 mm silicone foam closure	Bellco Glass	2004-00005	(none)
Shaking incubator for well plates	Kuhner	LT-X SMX1703	(none)
Shaking incubator for flasks and glass culture tubes	Thermo Fisher	Forma Model 440 Orbital Shaker	(none)
Methanol	Sigma-Aldrich	322415-1L	SHBJ0200
Acetone	Sigma-Aldrich	650501-1L	SHBJ0047
Dichloromethane	Sigma-Aldrich	270997-1L	SHBH7635

Supplementary Table 3--Comparison of measured and expected osmolality of 12 media solutions. The 8 left columns have the concentration of the components of 12 different media solutions. The right column reports the percent difference between the expected osmolality of the solution, and the measured osmolality of the solution.

Yeast extract (g L^{-1})	Tryptone (g L^{-1})	Glycerol (g L^{-1})	Glucose (g L^{-1})	Magnesium sulfate (g L^{-1})	Dipotassium phosphate (g L^{-1})	Potassium phosphate (g L^{-1})	Sodium chloride (g L^{-1})	Expected Osmolality (mmol kg^{-1})	Measured osmolality (mmol kg^{-1})	Percent difference
24	12	5	0	0	12.5	2.3	2.35	600	585	-2.5%
16	12	5	0	0	12.5	2.3	3.75	600	575	-4.2%
32	12	5	0	0	12.5	2.3	0.943	600	587	-2.2%
24	12	3	0	0	12.5	2.3	2.98	600	566	-5.7%
24	12	7	0	0	12.5	2.3	1.71	600	561	-6.5%
24	12	5	0	0.24	12.5	2.3	2.35	600	584	-2.7%
24	12	5	0	0	9.87	1.81	3.88	600	589	-1.8%
24	12	5	0	0	15.5	2.84	0.603	600	580	-3.3%
24	12	5	0	0	12.5	2.3	5.27	700	663	-5.3%
24	12	5	0	0	12.5	2.3	8.19	800	756	-5.5%
24	12	5	0	0	12.5	2.3	11.1	900	843	-6.3%
24	12	5	0	0	12.5	2.3	14	1000	939	-6.1%

Supplementary Table 4--Lycopene absorbance peaks and molar extinction coefficients[1].

	λ_1 (nm)	ε_1 (M^{-1} cm^{-1})	λ_2 (nm)	ε_2 (M^{-1} cm^{-1})	λ_3 (nm)	ε_3 (M^{-1} cm^{-1})
Methanol	444.0	---	469.5	---	501.5	---
Acetone	447.0	118,000	474.0	178,000	505.5	162,000
Dichloromethane	455.0	113,000	482.5	170,000	515.5	150,000

Supplementary Note 1

The linear model used to predict Group 2 responses from Group 1 responses (**Figure 1e**) was built using all main effect terms and significant two-factor interaction terms, as determined from examination of the normal plots **(Supplementary Fig. 3)**. The model summaries for the 3 responses (dcm, titer and yield) are shown below:

```
$dcm

Call:
lm.default(formula = response ~ (Yeast_extract + Yeast_extract_source +
    Tryptone + Glycerol + Magnesium_sulfate + pH + Buffer_capacity +
    Osmolality + Yeast_extract_source:Magnesium_sulfate + Shake_speed +
    Well_volume + Well_fill_volume + Well_bottom + Well_cover +
    Shake_speed:Well_volume + Shake_speed:Well_fill_volume +
    Time * Temperature + Antibiotic_conc + Inoculum_age + Inoculum_amount),
    data = .)

Residuals:
    Min      1Q  Median      3Q     Max
-2.0631 -0.8865  0.1113  0.7974  2.3435

Coefficients:
                                        Estimate Std. Error t value Pr(>|t|)
(Intercept)                            1.456e+01  6.700e+00   2.173 0.033026 *
Yeast_extract                          2.238e-01  5.317e-02   4.209  7.2e-05 ***
Yeast_extract_source                   1.594e+00  5.663e-01   2.815 0.006270 **
Tryptone                               9.424e-02  1.063e-01   0.886 0.378377
Glycerol                               4.199e-01  1.063e-01   3.949 0.000179 ***
Magnesium_sulfate                      2.237e+01  5.604e+00   3.991 0.000155 ***
pH                                    -8.391e-01  5.279e-01  -1.590 0.116266
Buffer_capacity                       -1.125e-02  1.063e-02  -1.058 0.293540
Osmolality                            -4.662e-03  2.127e-03  -2.192 0.031568 *
Shake_speed                            3.682e-03  4.308e-03   0.855 0.395597
Well_volume                            4.063e-01  1.754e-01   2.316 0.023353 *
Well_fill_volume                      -1.568e+01  6.578e+00  -2.384 0.019721 *
Well_bottom                           -3.890e-01  4.017e-01  -0.968 0.336105
Well_cover                             2.660e-01  4.017e-01   0.662 0.509984
Time                                  -2.242e-01  8.527e-02  -2.630 0.010418 *
Temperature                           -2.554e-01  1.331e-01  -1.919 0.058863 .
Antibiotic_conc                        2.874e+00  4.368e+00   0.658 0.512712
Inoculum_age                           8.147e-03  3.666e-03   2.222 0.029350 *
Inoculum_amount                        1.186e+01  1.092e+01   1.086 0.280925
Yeast_extract_source:Magnesium_sulfate -1.082e+01  3.544e+00  -3.054 0.003154 **
Shake_speed:Well_volume               -2.430e-04  4.658e-04  -0.522 0.603399
Shake_speed:Well_fill_volume           2.755e-02  1.747e-02   1.577 0.119013
Time:Temperature                       6.607e-03  2.532e-03   2.609 0.010994 *
---
Signif. codes:  0 '***' 0.001 '**' 0.01 '*' 0.05 '.' 0.1 ' ' 1

Residual standard error: 1.203 on 73 degrees of freedom
Multiple R-squared:  0.6634,     Adjusted R-squared:  0.562
```

```
F-statistic:  6.54 on 22 and 73 DF,  p-value: 4.797e-10


$titer

Call:
lm.default(formula = response ~ (Yeast_extract + Yeast_extract_source +
    Tryptone + Glycerol + Magnesium_sulfate + pH + Buffer_capacity +
    Osmolality + Yeast_extract_source:Magnesium_sulfate + Shake_speed +
    Well_volume + Well_fill_volume + Well_bottom + Well_cover +
    Shake_speed:Well_volume + Shake_speed:Well_fill_volume +
    Time * Temperature + Antibiotic_conc + Inoculum_age + Inoculum_amount),
    data = .)

Residuals:
    Min      1Q  Median      3Q     Max
-3.2789 -0.5774 -0.0163  0.5069  3.0601

Coefficients:
                                         Estimate Std. Error t value Pr(>|t|)
(Intercept)                             1.500e+00  6.713e+00   0.223   0.8239
Yeast_extract                           6.974e-02  5.327e-02   1.309   0.1945
Yeast_extract_source                    8.950e-01  5.674e-01   1.577   0.1190
Tryptone                                5.129e-02  1.065e-01   0.481   0.6316
Glycerol                                6.534e-01  1.065e-01   6.133 4.02e-08 ***
Magnesium_sulfate                       1.267e+01  5.615e+00   2.256   0.0271 *
pH                                     -3.759e-01  5.289e-01  -0.711   0.4795
Buffer_capacity                        -7.198e-03  1.065e-02  -0.676   0.5014
Osmolality                             -8.568e-04  2.131e-03  -0.402   0.6888
Shake_speed                             7.126e-03  4.317e-03   1.651   0.1031
Well_volume                             2.615e+00  1.758e-01  14.876  < 2e-16 ***
Well_fill_volume                       -4.019e+01  6.591e+00  -6.097 4.66e-08 ***
Well_bottom                             7.037e-02  4.025e-01   0.175   0.8617
Well_cover                             -8.703e-02  4.025e-01  -0.216   0.8294
Time                                   -1.320e-01  8.544e-02  -1.545   0.1266
Temperature                            -1.168e-01  1.333e-01  -0.876   0.3840
Antibiotic_conc                         3.141e+00  4.377e+00   0.718   0.4753
Inoculum_age                            2.198e-03  3.673e-03   0.598   0.5514
Inoculum_amount                         7.725e+00  1.094e+01   0.706   0.4824
Yeast_extract_source:Magnesium_sulfate -6.173e+00  3.551e+00  -1.738   0.0864 .
Shake_speed:Well_volume                -6.004e-03  4.667e-04 -12.866  < 2e-16 ***
Shake_speed:Well_fill_volume            1.328e-01  1.750e-02   7.590 8.29e-11 ***
Time:Temperature                        3.313e-03  2.537e-03   1.306   0.1956
---
Signif. codes:  0 '***' 0.001 '**' 0.01 '*' 0.05 '.' 0.1 ' ' 1

Residual standard error: 1.205 on 73 degrees of freedom
Multiple R-squared:  0.8452,     Adjusted R-squared:  0.7986
F-statistic: 18.12 on 22 and 73 DF,  p-value: < 2.2e-16



$yield
```

```
Call:
lm.default(formula = response ~ (Yeast_extract + Yeast_extract_source +
    Tryptone + Glycerol + Magnesium_sulfate + pH + Buffer_capacity +
    Osmolality + Yeast_extract_source:Magnesium_sulfate + Shake_speed +
    Well_volume + Well_fill_volume + Well_bottom + Well_cover +
    Shake_speed:Well_volume + Shake_speed:Well_fill_volume +
    Time * Temperature + Antibiotic_conc + Inoculum_age + Inoculum_amount),
    data = .)

Residuals:
     Min       1Q   Median       3Q      Max
-0.42809 -0.03943  0.00017  0.02578  0.32600

Coefficients:
                                        Estimate Std. Error t value Pr(>|t|)
(Intercept)                           -4.034e-01  6.280e-01  -0.642   0.5226
Yeast_extract                         -8.166e-03  4.983e-03  -1.639   0.1056
Yeast_extract_source                   5.368e-03  5.308e-02   0.101   0.9197
Tryptone                              -2.394e-03  9.967e-03  -0.240   0.8108
Glycerol                               5.372e-02  9.967e-03   5.390 8.27e-07 ***
Magnesium_sulfate                      1.287e-01  5.253e-01   0.245   0.8072
pH                                     1.696e-02  4.948e-02   0.343   0.7327
Buffer_capacity                       -2.823e-04  9.966e-04  -0.283   0.7778
Osmolality                             2.063e-04  1.993e-04   1.035   0.3042
Shake_speed                            8.418e-04  4.038e-04   2.084   0.0406 *
Well_volume                            2.749e-01  1.644e-02  16.722  < 2e-16 ***
Well_fill_volume                      -3.575e+00  6.166e-01  -5.799 1.59e-07 ***
Well_bottom                            4.816e-02  3.765e-02   1.279   0.2050
Well_cover                            -1.969e-02  3.765e-02  -0.523   0.6027
Time                                  -3.381e-04  7.993e-03  -0.042   0.9664
Temperature                            4.023e-03  1.247e-02   0.322   0.7480
Antibiotic_conc                        1.477e-01  4.094e-01   0.361   0.7193
Inoculum_age                          -2.746e-04  3.436e-04  -0.799   0.4268
Inoculum_amount                        1.057e-01  1.024e+00   0.103   0.9181
Yeast_extract_source:Magnesium_sulfate -7.483e-02  3.322e-01  -0.225   0.8224
Shake_speed:Well_volume               -6.637e-04  4.366e-05 -15.203  < 2e-16 ***
Shake_speed:Well_fill_volume           1.305e-02  1.637e-03   7.971 1.60e-11 ***
Time:Temperature                      -4.899e-05  2.373e-04  -0.206   0.8370
---
Signif. codes:  0 '***' 0.001 '**' 0.01 '*' 0.05 '.' 0.1 ' ' 1

Residual standard error: 0.1128 on 73 degrees of freedom
Multiple R-squared:  0.8654,     Adjusted R-squared:  0.8248
F-statistic: 21.33 on 22 and 73 DF,  p-value: < 2.2e-16
```

The same approach was applied to the RFP strains (**Supplementary Fig. 9e)**, with significant two-term interactions selected from examination of the normal plots **(Supplementary Fig. 11)**:

```
$dcm

Call:
lm.default(formula = response ~ (Yeast_extract + Yeast_extract_source +
    Tryptone + Glycerol + Magnesium_sulfate + pH + Buffer_capacity +
    Osmolality + Yeast_extract_source:Magnesium_sulfate + Shake_speed +
    Well_volume + Well_fill_volume + Well_bottom + Well_cover +
    Shake_speed:Well_volume + Shake_speed:Well_fill_volume +
    Well_volume:Well_fill_volume + Time * Temperature + Antibiotic_conc +
    Inoculum_age + Inoculum_amount), data = .)

Residuals:
     Min       1Q   Median       3Q      Max
-1.62377 -0.43149 -0.02039  0.38881  1.72481

Coefficients:
                                         Estimate Std. Error t value Pr(>|t|)
(Intercept)                             1.072e+01  3.856e+00   2.780 0.006933 **
Yeast_extract                           2.255e-01  3.062e-02   7.364 2.35e-10 ***
Yeast_extract_source2                   2.421e+00  3.265e-01   7.416 1.88e-10 ***
Tryptone                                1.044e-01  6.124e-02   1.705 0.092477 .
Glycerol                                4.245e-01  6.124e-02   6.932 1.47e-09 ***
Magnesium_sulfate                       1.396e+01  1.443e+00   9.672 1.18e-14 ***
pH                                     -6.856e-01  3.040e-01  -2.255 0.027170 *
Buffer_capacity                         3.106e-03  6.124e-03   0.507 0.613521
Osmolality                             -1.482e-03  1.225e-03  -1.210 0.230279
Shake_speed                             3.252e-03  2.533e-03   1.284 0.203367
Well_volume                             5.641e-01  1.222e-01   4.616 1.67e-05 ***
Well_fill_volume                       -1.751e+01  4.376e+00  -4.001 0.000151 ***
Well_bottom                            -8.490e-03  2.327e-01  -0.036 0.971001
Well_cover                             -2.215e-01  2.327e-01  -0.952 0.344281
Time                                   -2.698e-01  4.911e-02  -5.493 5.64e-07 ***
Temperature                            -2.218e-01  7.666e-02  -2.893 0.005042 **
Antibiotic_conc                         1.781e+00  1.258e+00   1.416 0.161228
Inoculum_age                            3.353e-03  2.112e-03   1.587 0.116830
Inoculum_amount                         9.208e+00  6.291e+00   1.464 0.147667
Yeast_extract_source2:Magnesium_sulfate -1.172e+01  2.041e+00  -5.743 2.07e-07 ***
Shake_speed:Well_volume                -1.209e-03  2.698e-04  -4.482 2.73e-05 ***
Shake_speed:Well_fill_volume            4.140e-02  1.012e-02   4.091 0.000110 ***
Well_volume:Well_fill_volume            4.454e-01  3.103e-01   1.435 0.155498
Time:Temperature                        7.912e-03  1.458e-03   5.427 7.35e-07 ***
---
Signif. codes:  0 '***' 0.001 '**' 0.01 '*' 0.05 '.' 0.1 ' ' 1

Residual standard error: 0.6928 on 72 degrees of freedom
Multiple R-squared:  0.8498,     Adjusted R-squared:  0.8018
F-statistic: 17.71 on 23 and 72 DF,  p-value: < 2.2e-16


$titer
```

```
Call:
lm.default(formula = response ~ (Yeast_extract + Yeast_extract_source +
    Tryptone + Glycerol + Magnesium_sulfate + pH + Buffer_capacity +
    Osmolality + Yeast_extract_source:Magnesium_sulfate + Shake_speed +
    Well_volume + Well_fill_volume + Well_bottom + Well_cover +
    Shake_speed:Well_volume + Shake_speed:Well_fill_volume +
    Well_volume:Well_fill_volume + Time * Temperature + Antibiotic_conc +
    Inoculum_age + Inoculum_amount), data = .)

Residuals:
     Min       1Q   Median       3Q      Max
-1.39758 -0.41205 -0.00763  0.35940  1.32117

Coefficients:
                                          Estimate Std. Error t value Pr(>|t|)
(Intercept)                             -3.454e+00  3.805e+00  -0.908 0.367109
Yeast_extract                            4.515e-02  3.021e-02   1.494 0.139430
Yeast_extract_source2                    2.043e+00  3.222e-01   6.342 1.76e-08 ***
Tryptone                                 2.814e-03  6.043e-02   0.047 0.962990
Glycerol                                 5.418e-01  6.043e-02   8.967 2.41e-13 ***
Magnesium_sulfate                        7.390e+00  1.424e+00   5.189 1.88e-06 ***
pH                                      -5.346e-02  3.000e-01  -0.178 0.859077
Buffer_capacity                          1.246e-02  6.043e-03   2.062 0.042837 *
Osmolality                              -1.691e-03  1.209e-03  -1.399 0.166022
Shake_speed                              4.274e-03  2.500e-03   1.710 0.091575 .
Well_volume                              1.366e+00  1.206e-01  11.325  < 2e-16 ***
Well_fill_volume                        -3.038e+01  4.318e+00  -7.034 9.56e-10 ***
Well_bottom                              2.620e-01  2.296e-01   1.141 0.257656
Well_cover                              -5.147e-01  2.296e-01  -2.241 0.028101 *
Time                                    -4.714e-03  4.846e-02  -0.097 0.922780
Temperature                             -1.552e-02  7.565e-02  -0.205 0.838054
Antibiotic_conc                         -2.186e-01  1.242e+00  -0.176 0.860758
Inoculum_age                            -5.635e-04  2.084e-03  -0.270 0.787682
Inoculum_amount                         -8.302e+00  6.208e+00  -1.337 0.185363
Yeast_extract_source2:Magnesium_sulfate -6.981e+00  2.014e+00  -3.466 0.000896 ***
Shake_speed:Well_volume                 -2.224e-03  2.663e-04  -8.354 3.35e-12 ***
Shake_speed:Well_fill_volume             9.901e-02  9.984e-03   9.917 4.20e-15 ***
Well_volume:Well_fill_volume            -1.884e+00  3.062e-01  -6.153 3.85e-08 ***
Time:Temperature                         1.002e-03  1.439e-03   0.697 0.488308
---
Signif. codes:  0 '***' 0.001 '**' 0.01 '*' 0.05 '.' 0.1 ' ' 1

Residual standard error: 0.6837 on 72 degrees of freedom
Multiple R-squared:  0.8939,     Adjusted R-squared:   0.86
F-statistic: 26.37 on 23 and 72 DF,  p-value: < 2.2e-16



$yield

Call:
lm.default(formula = response ~ (Yeast_extract + Yeast_extract_source +
    Tryptone + Glycerol + Magnesium_sulfate + pH + Buffer_capacity +
    Osmolality + Yeast_extract_source:Magnesium_sulfate + Shake_speed +
    Well_volume + Well_fill_volume + Well_bottom + Well_cover +
```

```
    Shake_speed:Well_volume + Shake_speed:Well_fill_volume +
    Well_volume:Well_fill_volume + Time * Temperature + Antibiotic_conc +
    Inoculum_age + Inoculum_amount), data = .)

Residuals:
      Min        1Q    Median        3Q       Max
-0.170431 -0.047111 -0.000763  0.043153  0.195332

Coefficients:
                                         Estimate Std. Error t value Pr(>|t|)
(Intercept)                            -4.339e-01  4.762e-01  -0.911 0.365283
Yeast_extract                          -1.126e-02  3.781e-03  -2.977 0.003958 **
Yeast_extract_source2                   2.277e-02  4.032e-02   0.565 0.574046
Tryptone                               -6.653e-03  7.563e-03  -0.880 0.381971
Glycerol                                2.630e-02  7.563e-03   3.477 0.000863 ***
Magnesium_sulfate                      -2.597e-01  1.783e-01  -1.457 0.149444
pH                                      5.112e-02  3.755e-02   1.362 0.177591
Buffer_capacity                         1.326e-03  7.563e-04   1.753 0.083804 .
Osmolality                             -7.826e-05  1.512e-04  -0.517 0.606435
Shake_speed                             5.939e-04  3.128e-04   1.899 0.061634 .
Well_volume                             1.247e-01  1.509e-02   8.265 4.92e-12 ***
Well_fill_volume                       -2.928e+00  5.404e-01  -5.419 7.59e-07 ***
Well_bottom                             3.246e-02  2.874e-02   1.129 0.262514
Well_cover                             -3.858e-02  2.874e-02  -1.342 0.183715
Time                                    1.417e-02  6.065e-03   2.337 0.022243 *
Temperature                             7.915e-03  9.467e-03   0.836 0.405896
Antibiotic_conc                        -1.550e-01  1.554e-01  -0.998 0.321751
Inoculum_age                           -2.869e-04  2.609e-04  -1.100 0.275044
Inoculum_amount                        -1.435e+00  7.770e-01  -1.847 0.068856 .
Yeast_extract_source2:Magnesium_sulfate  1.590e-01  2.521e-01   0.631 0.530309
Shake_speed:Well_volume                -2.142e-04  3.332e-05  -6.429 1.23e-08 ***
Shake_speed:Well_fill_volume            8.980e-03  1.250e-03   7.187 4.99e-10 ***
Well_volume:Well_fill_volume           -1.765e-01  3.832e-02  -4.606 1.73e-05 ***
Time:Temperature                       -3.292e-04  1.801e-04  -1.828 0.071642 .
---
Signif. codes:  0 '***' 0.001 '**' 0.01 '*' 0.05 '.' 0.1 ' ' 1

Residual standard error: 0.08556 on 72 degrees of freedom
Multiple R-squared:  0.8201,     Adjusted R-squared:  0.7627
F-statistic: 14.27 on 23 and 72 DF,  p-value: < 2.2e-16
```

Supplementary Note 2

Description of MIEO v0.1.0

The key words “MUST”, “MUST NOT”, “REQUIRED”, “SHALL”, “SHALL NOT”, “SHOULD”, “SHOULD NOT”, “RECOMMENDED”, “MAY”, and “OPTIONAL” in this document are to be interpreted as described in RFC 2119.[3]

There are 9 categories of factors in the Minimum Information Standard for Engineering Organism Experiments (MIEO) v0.1.0. Categories are either REQUIRED (R), meaning that they must be specified, or OPTIONAL (O), meaning that the user can decide whether it is appropriate to specify them. Within each category, the factors to be specified are left up to the individual user, because every situation will call for a different set of factors. The descriptions of the categories below, subsequent example, and the table of factors after that should provide indicate what factors should be included. The categories are:

1) **Media Components** (R)--This category MUST include the identity and concentration of each component in the media. For complex media components of undefined composition, such as yeast extract, manufacturer and lot number SHOULD also be provided.

2) **Media Properties** (O)--This category MAY include any properties of the media, such as pH, buffer capacity, or osmolality. This category is OPTIONAL, because these values can be derived from the media components. Including information about media properties is helpful.

3) **Container Geometry** (R)--This category MUST include any factors relating to the physical geometry of the container in which the cells are grown. For microtiter plates, this includes the number of wells per plate, the shape of the sides of the well, the shape of the bottom of the well, the volume of the well, the fraction of the well volume filled with media, and the cover of the well. For shake flasks, this includes the presence or absence of baffles, the number of baffles, the dimensions of the baffles, the volume of the flask, and the cover of the flask.

4) **Container Shaking** (R)--This category MUST include any factors relating to how the container was shaken during the growth of the cells. This includes the speed, diameter, and mode (orbital or linear) of the shaking.

5) **Time** (R)--This category MUST include the length of time for which the cells were grown, from inoculation until the assay. If the growth is interrupted for intermediate sampling, that MUST be indicated as well.

6) **Environment** (R)--This category SHOULD include all factors related to the environment outside of the growth container. Temperature is the most important factor, and MUST be included. Additional factors that MAY be included are relative humidity, barometric pressure, etc.

7) **Inoculum** (R)--This category MUST include factors related to the cell inoculum used to seed the culture, including the concentration of cells in the inoculum, the age of the inoculum, the phase in the cell cycle of the cells in the inoculum (e.g., exponential or stationary).

8) **Selective Agents** (O)--This category SHOULD include any factors related to additives to the media used to select for or against the growth of cells in the culture, such as the identity and concentration of antibiotics used in bacterial cultures or counter-selecting agents used in yeast cultures. This category is OPTIONAL because not all cultures use selective agents.

9) **Inducers** (O)--This category SHOULD include any factors related to additives to the media used to induce the expression of genes from cells in the culture, such as the identity and concentration of inducers, as well as the time at which they were added to the culture. This category is OPTIONAL because not all cultures use inducers.

Supplementary References

1. Takehara, M. *et al.* Characterization and thermal isomerization of (all-E)-lycopene. *J. Agric. Food Chem.* **62,** 264–269 (2014).
2. Hecht, A., Endy, D., Salit, M. & Munson, M. S. When wavelengths collide: bias in cell abundance measurements due to expressed fluorescent proteins. *ACS Synth. Biol.* **5,** 1024–1027 (2016).
3. Bradner, S. RFC 2119 - Key words for use in RFCs to Indicate Requirement Levels. (1997). at <https://tools.ietf.org/html/rfc2119>
4. Fox, J. *Applied Regression Analysis and Generalized Linear Models*. (SAGE Publications, 2015).
5. Neidhardt, F. C., Ingraham, J. L. & Schaechter, M. *Physiology of the Bacterial Cell: A Molecular Approach*. (Sinauer Associates Inc, 1990).
6. Studier, F. W. Protein production by auto-induction in high density shaking cultures. *Protein Expr. Purif.* **41,** 207–234 (2005).
7. Cunningham, F. X., Lee, H. & Gantt, E. Carotenoid biosynthesis in the primitive red alga Cyanidioschyzon merolae. *Eukaryotic Cell* **6,** 533–545 (2007).
