# Supplementary material for: A minimum information standard for reproducing bench-scale bacterial cell growth and productivity
Source: Commun Biol. 2018 Dec 6;1:219. doi: 10.1038/s42003-018-0220-6 (PMC6283831; doi:10.1038/s42003-018-0220-6)
Supplement: Supplementary file 4 — Supplementary Data 3 [file 42003_2018_220_MOESM4_ESM.rtf]

LOCUS       Exported                9476 bp ds-DNA     circular SYN 10-JAN-2018DEFINITION  Contains 3 genes (crtE, crtI, and crtB) of the carotenoid pathway             gene cluster of Erwinia herbicola (Pantoea agglomerans) Eho10 and             thereby produces lycopene in Escherichia coli.ACCESSION   .VERSION     .KEYWORDS    pAC-LYCSOURCE      synthetic DNA construct  ORGANISM  synthetic DNA constructREFERENCE   1  (bases 1 to 9476)  AUTHORS   Cunningham FX Jr, Sun Z, Chamovitz D, Hirschberg J, Gantt E  TITLE     Molecular structure and enzymatic function of lycopene cyclase from             the cyanobacterium Synechococcus sp strain PCC7942.  JOURNAL   Plant Cell. 1994 Aug;6(8):1107-21.  PUBMED    7919981REFERENCE   2  (bases 1 to 9476)  AUTHORS   .  TITLE     Direct Submission  JOURNAL   Exported Jan 10, 2018 from SnapGene Server 1.1.58            http://www.snapgene.comFEATURES             Location/Qualifiers     source          1..9476                     /organism="synthetic DNA construct"                     /mol_type="other DNA"     primer_bind     134..153                     /label=CAT-R                     /note="Chloramphenicol resistance gene, reverse primer"     promoter        complement(220..322)                     /label=cat promoter                     /note="promoter of the E. coli cat gene encoding                      chloramphenicol acetyltransferase"     primer_bind     complement(714..731)                     /label=L4440                     /note="L4440 vector, forward primer"     rep_origin      complement(848..1393)                     /direction=LEFT                     /label=p15A ori                     /note="Plasmids containing the medium-copy-number p15A                      origin of replication can be propagated in E. coli cells                      that contain a second plasmid with the ColE1 origin.                     "     primer_bind     complement(928..947)                     /label=pBR322ori-F                     /note="pBR322 origin, forward primer"     promoter        1505..1533                     /label=tet promoter                     /note="E. coli promoter for tetracycline efflux protein                      gene"     primer_bind     complement(7164..7183)                     /label=pBRrevBam                     /note="pBR322 vectors, tet region, downstream of BamHI,                      reverse primer"     CDS             complement(9036..219)                     /codon_start=1                     /gene="cat"                     /product="chloramphenicol acetyltransferase"                     /label=CmR                     /note="confers resistance to chloramphenicol"                     /translation="MEKKITGYTTVDISQWHRKEHFEAFQSVAQCTYNQTVQLDITAFL                     KTVKKNKHKFYPAFIHILARLMNAHPEFRMAMKDGELVIWDSVHPCYTVFHEQTETFSS                     LWSEYHDDFRQFLHIYSQDVACYGENLAYFPKGFIENMFFVSANPWVSFTSFDLNVANM                     DNFFAPVFTMGKYYTQGDKVLMPLAIQVHHAVCDGFHVGRMLNELQQYCDEWQGGA"ORIGIN        1 gaattccgga tgagcattca tcaggcgggc aagaatgtga ataaaggccg gataaaactt       61 gtgcttattt ttctttacgg tctttaaaaa ggccgtaata tccagctgaa cggtctggtt      121 ataggtacat tgagcaactg actgaaatgc ctcaaaatgt tctttacgat gccattggga      181 tatatcaacg gtggtatatc cagtgatttt tttctccatt ttagcttcct tagctcctga      241 aaatctcgat aactcaaaaa atacgcccgg tagtgatctt atttcattat ggtgaaagtt      301 ggaacctctt acgtgccgat caacgtctca ttttcgccaa aagttggccc agggcttccc      361 ggtatcaaca gggacaccag gatttattta ttctgcgaag tgatcttccg tcacaggtat      421 ttattcggcg caaagtgcgt cgggtgatgc tgccaactta ctgatttagt gtatgatggt      481 gtttttgagg tgctccagtg gcttctgttt ctatcagctg tccctcctgt tcagctactg      541 acggggtggt gcgtaacggc aaaagcaccg ccggacatca gcgctagcgg agtgtatact      601 ggcttactat gttggcactg atgagggtgt cagtgaagtg cttcatgtgg caggagaaaa      661 aaggctgcac cggtgcgtca gcagaatatg tgatacagga tatattccgc ttcctcgctc      721 actgactcgc tacgctcggt cgttcgactg cggcgagcgg aaatggctta cgaacggggc      781 ggagatttcc tggaagatgc caggaagata cttaacaggg aagtgagagg gccgcggcaa      841 agccgttttt ccataggctc cgcccccctg acaagcatca cgaaatctga cgctcaaatc      901 agtggtggcg aaacccgaca ggactataaa gataccaggc gtttccccct ggcggctccc      961 tcgtgcgctc tcctgttcct gcctttcggt ttaccggtgt cattccgctg ttatggccgc     1021 gtttgtctca ttccacgcct gacactcagt tccgggtagg cagttcgctc caagctggac     1081 tgtatgcacg aaccccccgt tcagtccgac cgctgcgcct tatccggtaa ctatcgtctt     1141 gagtccaacc cggaaagaca tgcaaaagca ccactggcag cagccactgg taattgattt     1201 agaggagtta gtcttgaagt catgcgccgg ttaaggctaa actgaaagga caagttttgg     1261 tgactgcgct cctccaagcc agttacctcg gttcaaagag ttggtagctc agagaacctt     1321 cgaaaaaccg ccctgcaagg cggttttttc gttttcagag caagagatta cgcgcagacc     1381 aaaacgatct caagaagatc atcttattaa tcagataaaa tatttctaga tttcagtgca     1441 atttatctct tcaaatgtag cacctgaagt cagccccata cgatataagt tgtaattctc     1501 atgtttgaca gcttatcatc gataagcttt aatgcggtag tttatcacag ttaaattgct     1561 aacgcagtca ggcaccgtgt atgaaatcta acaatgcgct catcgtcatc ctcggcaccg     1621 tcaccctgga tgctgtaggc ataggcttgg ttatgccggt actgccgggc ctcttgcggg     1681 atatcgtcca ttccgacagc atcgccagtc actatggcgt gctgctagcg ctatatgcgt     1741 tgatgcaatt tctatgcgca cccgttctcg gagcactgtc cgaccgcttt ggccgccgcc     1801 cagtcctgct cgcttcgcta cttggagcca ctatcgacta cgcgatcatg gcgaccacac     1861 ccgtcctgtg gatctaaagg cacagcgtct catgcttcgc acaatgtaaa actgcttcag     1921 aacctggcga gagctatccg cgcggtctac ggttaactga tactaaaaga caattcagcg     1981 ggtaaccttg caatggtgag tggcagtaaa gcgggcgttt cgcctcatcg cgaaatagaa     2041 gtaatgagac aatccattga cgatcacctg gctggcctgt tacctgaaac cgacagccag     2101 gatatcgtca gccttgcgat gcgtgaaggc gtcatggcac ccggtaaacg gatccgtccg     2161 ctgctgatgc tgctggccgc ccgcgacctc cgctaccagg gcagtatgcc tacgctgctc     2221 gatctcgcct gcgccgttga actgacccat accgcgtcgc tgatgctcga cgacatgccc     2281 tgcatggaca acgccgagct gcgccgcggt cagcccacta cccacaaaaa atttggtgag     2341 agcgtggcga tccttgcctc cgttgggctg ctctctaaag cctttggtct gatcgccgcc     2401 accggcgatc tgccggggga gaggcgtgcc caggcggtca acgagctctc taccgccgtg     2461 ggcgtgcagg gcctggtact ggggcagttt cgcgatctta acgatgccgc cctcgaccgt     2521 acccctgacg ctatcctcag caccaaccac ctcaagaccg gcattctgtt cagcgcgatg     2581 ctgcagatcg tcgccattgc ttccgcctcg tcgccgagca cgcgagagac gctgcacgcc     2641 ttcgccctcg acttcggcca ggcgtttcaa ctgctggacg atctgcgtga cgatcacccg     2701 gaaaccggta aagatcgcaa taaggacgcg ggaaaatcga cgctggtcaa ccggctgggc     2761 gcagacgcgg cccggcaaaa gctgcgcgag catattgatt ccgccgacaa acacctcact     2821 tttgcctgtc cgcagggcgg cgccatccga cagtttatgc atctgtggtt tggccatcac     2881 cttgccgact ggtcaccggt catgaaaatc gcctgatacc gcccttttgg gttcaagcag     2941 tacataacga tggaaccaca ttacaggagt agtgatgaat gaaggacgag cgccttgttc     3001 agcgtaagaa cgatcatctg gatatcgttc tcgacccccg tcgcgccgta actcaggcta     3061 gcgcaggttt tgagcgctgg cgctttaccc actgcgccct gccagagctg aattttagcg     3121 acatcacgct ggaaaccacc ttcctgaatc ggcagctaca ggctccgctg ctgatcagct     3181 ccatgaccgg cggcgttgag cgctcgcgcc atatcaaccg ccacctcgcc gaggcggcgc     3241 aggtgctaaa aattgcgatg ggggtgggct cccagcgcgt cgccattgag agcgacgcgg     3301 gcttagggct ggataaaacc ctgcggcagc tggctccgga cgtgccgctg ctggcgaacc     3361 tcggcgcggc gcagctgacc ggcagaaaag gtattgatta cgcccgacgg gccgtggaga     3421 tgatcgaggc ggatgcgctg attgtgcacc taaacccgct gcaggaggcg ctacagcccg     3481 gcggcgatcg cgactggcgc ggacggctgg cggctattga aactctggtc cgcgagctgc     3541 ccgttccgct ggtggtgaaa gaggtgggag ccggtatctc ccgaaccgtg gccgggcagc     3601 tgatcgatgc cggcgttacc gtgattgacg tcgcgggcgc gggcggcacc agctgggccg     3661 ccgttgaagg cgagcgggcg gccaccgagc agcagcgcag cgtggccaac gtctttgccg     3721 actgggggat ccccaccgct gaggcgctgg ttgacattgc cgaggcctgg ccgcagatgc     3781 cccttattgc ctcgggaatg cgggctgggc tatttcaccc taccactggc tattcgctgc     3841 cgctggcggt ggcccttgcc gacgcgattg ccgacagccc gcggctgggc agcgttccgc     3901 tctatcagct cacccggcag tttgccgaac gccactggcg caggcaggga ttcttccgcc     3961 tgctgaaccg gatgcttttc ctggccgggc gcgaggagaa ccgctggcgg gtgatgcagc     4021 gcttttatgg gctgccggag cccaccgtag agcgctttta cgccggtcgg ctctctctct     4081 ttgataaggc ccgcattttg acgggcaagc caccggttcc gctgggcgaa gcctggcggg     4141 cggcgctgaa ccattttcct gacagacgag ataaaggatg aaaaaaaccg ttgtgattgg     4201 cgcaggcttt ggtggcctgg cgctggcgat tcgcctgcag gcggcaggga tcccaaccgt     4261 actgctggag cagcgggaca agcccggcgg tcgggcctac gtctggcatg accagggctt     4321 tacctttgac gccgggccga cggtgatcac cgatcctacc gcgcttgagg cgctgttcac     4381 cctggccggc aggcgcatgg aggattacgt caggctgctg ccggtaaaac ccttctaccg     4441 actctgctgg gagtccggga agaccctcga ctatgctaac gacagcgccg agcttgaggc     4501 gcagattacc cagttcaacc cccgcgacgt cgagggctac cggcgctttc tggcttactc     4561 ccaggcggta ttccaggagg gatatttgcg cctcggcagc gtgccgttcc tctcttttcg     4621 cgacatgctg cgcgccgggc cgcagctgct taagctccag gcgtggcaga gcgtctacca     4681 gtcggtttcg cgctttattg aggatgagca tctgcggcag gccttctcgt tccactccct     4741 gctggtaggc ggcaacccct tcaccacctc gtccatctac accctgatcc acgcccttga     4801 gcgggagtgg ggggtctggt tccctgaggg cggcaccggg gcgctggtga acggcatggt     4861 gaagctgttt accgatctgg gcggggagat cgaactcaac gcccgggtcg aagagctggt     4921 ggtggccgat aaccgcgtaa gccaggtccg gctggcggat ggtcggatct ttgacaccga     4981 cgccgtagcc tcgaacgctg acgtggtgaa cacctataaa aagctgctcg gccaccatcc     5041 ggtggggcag aagcgggcgg cagcgctgga gcgcaagagc atgagcaact cgctgtttgt     5101 gctctacttc ggcctgaacc agcctcattc ccagctggcg caccatacca tctgttttgg     5161 tccccgctac cgggagctga tcgacgagat ctttaccggc agcgcgctgg cggatgactt     5221 ctcgctctac ctgcactcgc cctgcgtgac cgatccctcg ctcgcgcctc ccggctgcgc     5281 cagcttctac gtgctggccc cggtgccgca tcttggcaac gcgccgctgg actgggcgca     5341 ggaggggccg aagctgcgcg accgcatctt tgactacctt gaagagcgct atatgcccgg     5401 cctgcgtagc cagctggtga cccagcggat ctttaccccg gcagacttcc acgacacgct     5461 ggatgcgcat ctgggatcgg ccttctccat cgagccgctg ctgacccaaa gcgcctggtt     5521 ccgcccgcac aaccgcgaca gcgacattgc caacctctac ctggtgggcg caggtactca     5581 ccctggggcg ggcattcctg gcgtagtggc ctcggcgaaa gccaccgcca gcctgatgat     5641 tgaggatctg caatgagcca accgccgctg cttgaccacg ccacgcagac catggccaac     5701 ggctcgaaaa gttttgccac cgctgcgaag ctgttcgacc cggccacccg ccgtagcgtg     5761 ctgatgctct acacctggtg ccgccactgc gatgacgtca ttgacgacca gacccacggc     5821 ttcgccagcg aggccgcggc ggaggaggag gccacccagc gcctggcccg gctgcgcacg     5881 ctgaccctgg cggcgtttga aggggccgag atgcaggatc cggccttcgc tgcctttcag     5941 gaggtggcgc tgacccacgg tattacgccc cgcatggcgc tcgatcacct cgacggcttt     6001 gcgatggacg tggctcagac ccgctatgtc acctttgagg atacgctgcg ctactgctat     6061 cacgtggcgg gcgtggtggg tctgatgatg gccagggtga tgggcgtgcg ggatgagcgg     6121 gtgctggatc gcgcctgcga tctggggctg gccttccagc tgacgaatat cgcccgggat     6181 attattgacg atgcggctat tgaccgctgc tatctgcccg ccgagtggct gcaggatgcc     6241 gggctgaccc cggagaacta tgccgcgcgg gagaatcggg ccgcgctggc gcgggtggcg     6301 gagcggctta ttgatgccgc agagccgtac tacatctcct cccaggccgg gctacacgat     6361 ctgccgccgc gctgcgcctg ggcgatcgcc accgcccgca gcgtctaccg ggagatcggt     6421 attaaggtaa aagcggcggg aggcagcgcc tgggatcgcc gccagcacac cagcaaaggt     6481 gaaaaaattg ccatgctgat ggcggcaccg gggcaggtta ttcgggcgaa gacgacgagg     6541 gtgacgccgc gtccggccgg tctttggcag cgtcccgttt aggcgggcgg ccatgacgtt     6601 cacgcaggat cgcctgtagg tcggcaggct tgcgggcgta aataaaaccg aaggagacgc     6661 agccctcccg gccgcgcacc gcgtggtgca ggcggtgggc gacgtagagc cgcttcaggt     6721 agccccggcg cgggatcccg cctcgccgtg ctgtccggtc tcaacctgat ccgccagaat     6781 cgagccaacg ggatcggcca gcacgaattc ggtatgcgga gagtgtttgg caaaccatgc     6841 ctgcaagcca cccagcgtgc cgccggagcc aacgcctact accacggcat caaccctgcc     6901 tgccagctgg tcgaacagct ccggtgcggt ggtggtggcg tgcgccagcg ggttggccgg     6961 gttagagaac tgatcaatat agtaagcacc cggcgtctcc tctgccaggc ggcgggcgta     7021 gtcctgatag tactccgggt ggccctttgt cacgtcggag cgggtcaggc gcacatcaac     7081 acccagcgca cgcaggtggt agatcctcta cgccggacgc atcgtggccg gcatcaccgg     7141 cgccacaggt gcggttgctg gcgcctatat cgccgacatc accgatgggg aagatcgggc     7201 tcgccacttc gggctcatga gcgcttgttt cggcgtgggt atggtggcag gccccgtggc     7261 cgggggactg ttgggcgcca tctccttgca tgcaccattc cttgcggcgg cggtgctcaa     7321 cggcctcaac ctactactgg gctgcttcct aatgcaggag tcgcataagg gagagcgtcg     7381 accgatgccc ttgagagcct tcaacccagt cagctccttc cggtgggcgc ggggcatgac     7441 tatcgtcgcc gcacttatga ctgtcttctt tatcatgcaa ctcgtaggac aggtgccggc     7501 agcgctctgg gtcattttcg gcgaggaccg ctttcgctgg agcgcgacga tgatcggcct     7561 gtcgcttgcg gtattcggaa tcttgcacgc cctcgctcaa gccttcgtca ctggtcccgc     7621 caccaaacgt ttcggcgaga agcaggccat tatcgccggc atggcggccg acgcgctggg     7681 ctacgtcttg ctggcgttcg cgacgcgagg ctggatggcc ttccccatta tgattcttct     7741 cgcttccggc ggcatcggga tgcccgcgtt gcaggccatg ctgtccaggc aggtagatga     7801 cgaccatcag ggacagcttc aaggatcgct cgcggctctt accagcctaa cttcgatcac     7861 tggaccgctg atcgtcacgg cgatttatgc cgcctcggcg agcacatgga acgggttggc     7921 atggattgta ggcgccgccc tataccttgt ctgcctcccc gcgttgcgtc gcggtgcatg     7981 gagccgggcc acctcgacct gaatggaagc cggcggcacc tcgctaacgg attcaccact     8041 ccaagaattg gagccaatca attcttgcgg agaactgtga atgcgcaaac caacccttgg     8101 cagaacatat ccatcgcgtc cgccatctcc agcagccgca cgcggcgcat ctcgggcagc     8161 gttgggtcct ggccacgggt gcgcatgatc gtgctcctgt cgttgaggac ccggctaggc     8221 tggcggggtt gccttactgg ttagcagaat gaatcaccga tacgcgagcg aacgtgaagc     8281 gactgctgct gcaaaacgtc tgcgacctga gcaacaacat gaatggtctt cggtttccgt     8341 gtttcgtaaa gtctggaaac gcggaagtcc cctacgtgct gctgaagttg cccgcaacag     8401 agagtggaac caaccggtga taccacgata ctatgactga gagtcaacgc catgagcggc     8461 ctcatttctt attctgagtt acaacagtcc gcaccgctgt ccggtagctc cttccggtgg     8521 gcgcggggca tgactatcgt cgccgcactt atgactgtct tctttatcat gcaactcgta     8581 ggacaggtgc cggcagcgcc caacagtccc ccggccacgg ggcctgccac catacccacg     8641 ccgaaacaag cgccctgcac cattatgttc cggatctgca tcgcaggatg ctgctggcta     8701 ccctgtggaa cacctacatc tgtattaacg aagcgctaac cgtttttatc aggctctggg     8761 aggcagaata aatgatcata tcgtcaatta ttacctccac ggggagagcc tgagcaaact     8821 ggcctcaggc atttgagaag cacacggtca cactgcttcc ggtagtcaat aaaccggtaa     8881 accagcaata gacataagcg gctatttaac gaccctgccc tgaaccgacg accgggtcga     8941 atttgctttc gaatttctgc cattcatccg cttattatca cttattcagg cgtagcacca     9001 ggcgtttaag ggcaccaata actgccttaa aaaaattacg ccccgccctg ccactcatcg     9061 cagtactgtt gtaattcatt aagcattctg ccgacatgga agccatcaca gacggcatga     9121 tgaacctgaa tcgccagcgg catcagcacc ttgtcgcctt gcgtataata tttgcccatg     9181 gtgaaaacgg gggcgaagaa gttgtccata ttggccacgt ttaaatcaaa actggtgaaa     9241 ctcacccagg gattggctga gacgaaaaac atattctcaa taaacccttt agggaaatag     9301 gccaggtttt caccgtaaca cgccacatct tgcgaatata tgtgtagaaa ctgccggaaa     9361 tcgtcgtggt attcactcca gagcgatgaa aacgtttcag tttgctcatg gaaaacggtg     9421 taacaagggt gaacactatc ccatatcacc agctcaccgt ctttcattgc catacg//LOCUS       Exported                3020 bp ds-DNA     circular SYN 10-JAN-2018DEFINITION  BIOFAB RFP reporter plasmid for measuring promoter 14 + BCD16             efficiency..ACCESSION   .VERSION     .KEYWORDS    pFAB3992SOURCE      synthetic DNA construct  ORGANISM  synthetic DNA constructREFERENCE   1  (bases 1 to 3020)  AUTHORS   Mutalik VK, Guimaraes JC, Cambray G, Lam C, Christoffersen MJ, Mai             QA, Tran AB, Paull M, Keasling JD, Arkin AP, Endy D  TITLE     Precise and reliable gene expression via standard transcription and             translation initiation elements.  JOURNAL   Nat Methods. 2013 Apr;10(4):354-60. doi: 10.1038/nmeth.2404. Epub             2013 Mar 10.  PUBMED    23474465REFERENCE   2  (bases 1 to 3020)  AUTHORS   .  TITLE     Direct Submission  JOURNAL   Exported Jan 10, 2018 from SnapGene Server 1.1.58            http://www.snapgene.comFEATURES             Location/Qualifiers     source          1..3020                     /organism="synthetic DNA construct"                     /mol_type="other DNA"     terminator      97..191                     /label=lambda t0 terminator                     /note="transcription terminator from phage lambda"     CDS             complement(222..1016)                     /codon_start=1                     /gene="aph(3')-II (or nptII)"                     /product="aminoglycoside phosphotransferase from Tn5"                     /label=NeoR/KanR                     /note="confers resistance to neomycin, kanamycin, and G418                      (Geneticin(R))"                     /translation="MIEQDGLHAGSPAAWVERLFGYDWAQQTIGCSDAAVFRLSAQGRP                     VLFVKTDLSGALNELQDEAARLSWLATTGVPCAAVLDVVTEAGRDWLLLGEVPGQDLLS                     SHLAPAEKVSIMADAMRRLHTLDPATCPFDHQAKHRIERARTRMEAGLVDQDDLDEEHQ                     GLAPAELFARLKARMPDGEDLVVTHGDACLPNIMVENGRFSGFIDCGRLGVADRYQDIA                     LATRDIAEELGGEWADRFLVLYGIAAPDSQRIAFYRLLDEFF"     primer_bind     complement(333..352)                     /label=Neo-F                     /note="Neomycin resistance gene, forward primer"     primer_bind     943..962                     /label=Neo-R                     /note="Neomycin resistance gene, reverse primer"     promoter        1336..1365                     /label=trc promoter                     /note="strong E. coli promoter; hybrid between the trp and                      lac UV5 promoters"     CDS             1457..2134                     /codon_start=1                     /product="monomeric derivative of DsRed (Campbell et al.,                      2002)"                     /label=mRFP1                     /translation="MASSEDVIKEFMRFKVRMEGSVNGHEFEIEGEGEGRPYEGTQTAK                     LKVTKGGPLPFAWDILSPQFQYGSKAYVKHPADIPDYLKLSFPEGFKWERVMNFEDGGV                     VTVTQDSSLQDGEFIYKVKLRGTNFPSDGPVMQKKTMGWEASTERMYPEDGALKGEIKM                     RLKLKDGGHYDAEVKTTYMAKKPVQLPGAYKTDIKLDITSHNEDYTIVEQYERAEGRHS                     TGA"     terminator      2167..2238                     /label=rrnB T1 terminator                     /note="transcription terminator T1 from the E. coli rrnB                      gene"     terminator      2254..2281                     /label=T7Te terminator                     /note="phage T7 early transcription terminator"     primer_bind     complement(2324..2341)                     /label=L4440                     /note="L4440 vector, forward primer"     rep_origin      complement(2458..3003)                     /direction=LEFT                     /label=p15A ori                     /note="Plasmids containing the medium-copy-number p15A                      origin of replication can be propagated in E. coli cells                      that contain a second plasmid with the ColE1 origin.                     "     primer_bind     complement(2538..2557)                     /label=pBR322ori-F                     /note="pBR322 origin, forward primer"ORIGIN        1 tcagataaaa tatttctaga tttcagtgca atttatctct tcaaatgtag cacctgaagt       61 cagccccata cgatataagt tgttactagt gcttggattc tcaccaataa aaaacgcccg      121 gcggcaaccg agcgttctga acaaatccag atggagttct gaggtcatta ctggatctat      181 caacaggagt ccaagcgagc tctcgaaccc cagagtcccg ctcagaagaa ctcgtcaaga      241 aggcgataga aggcgatgcg ctgcgaatcg ggagcggcga taccgtaaag cacgaggaag      301 cggtcagccc attcgccgcc aagctcttca gcaatatcac gggtagccaa cgctatgtcc      361 tgatagcggt ccgccacacc cagccggcca cagtcgatga atccagaaaa gcggccattt      421 tccaccatga tattcggcaa gcaggcatcg ccatgggtca cgacgagatc ctcgccgtcg      481 ggcatgcgcg ccttgagcct ggcgaacagt tcggctggcg cgagcccctg atgctcttcg      541 tccagatcat cctgatcgac aagaccggct tccatccgag tacgtgctcg ctcgatgcga      601 tgtttcgctt ggtggtcgaa tgggcaggta gccggatcaa gcgtatgcag ccgccgcatt      661 gcatcagcca tgatggatac tttctcggca ggagcaaggt gagatgacag gagatcctgc      721 cccggcactt cgcccaatag cagccagtcc cttcccgctt cagtgacaac gtcgagcaca      781 gctgcgcaag gaacgcccgt cgtggccagc cacgatagcc gcgctgcctc gtcctgcagt      841 tcattcaggg caccggacag gtcggtcttg acaaaaagaa ccgggcgccc ctgcgctgac      901 agccggaaca cggcggcatc agagcagccg attgtctgtt gtgcccagtc atagccgaat      961 agcctctcca cccaagcggc cggagaacct gcgtgcaatc catcttgttc aatcatgcga     1021 aacgatcctc atcctgtctc ttgatcagat catgatcccc tgcgccatca gatccttggc     1081 ggcaagaaag ccatccagtt tactttgcag ggcttcccaa ccttaccaga gggcgcccca     1141 gctggcaatt ccgacgtcga attcaaaaga tcttaagtaa gtaagagtat acgtatatcg     1201 gctaataacg tattaaggcg cttcggcgcc tttttttatg ggggtatttt catcccaatc     1261 cacacgtcca acgcacagca aacaccacgt cgaccctatc agctgcgtgc tttctatgag     1321 tcgttgctgc ataacttgac aattaatcat ccggctcgta taatgtgtgg agggcccaag     1381 ttcacttaaa aaggagatca acaatgaaag caattttcgt actgaaacat cttaatcatg     1441 cttaggagtc tttctaatgg cgagtagcga agacgttatc aaagagttca tgcgtttcaa     1501 agttcgtatg gaaggttccg ttaacggtca cgagttcgaa atcgaaggtg aaggtgaagg     1561 tcgtccgtac gaaggtaccc agaccgctaa actgaaagtt accaaaggtg gtccgctgcc     1621 gttcgcttgg gacatcctgt ccccgcagtt ccagtacggt tccaaagctt acgttaaaca     1681 cccggctgac atcccggact acctgaaact gtccttcccg gaaggtttca aatgggaacg     1741 tgttatgaac ttcgaagacg gtggtgttgt taccgttacc caggactcct ccctgcaaga     1801 cggtgagttc atctacaaag ttaaactgcg tggtaccaac ttcccgtccg acggtccggt     1861 tatgcagaaa aaaaccatgg gttgggaagc ttccaccgaa cgtatgtacc cggaagacgg     1921 tgctctgaaa ggtgaaatca aaatgcgtct gaaactgaaa gacggtggtc actacgacgc     1981 tgaagttaaa accacctaca tggctaaaaa accggttcag ctgccgggtg cttacaaaac     2041 cgacatcaaa ctggacatca cctcccacaa cgaagactac accatcgttg aacagtacga     2101 acgtgctgaa ggtcgtcact ccaccggtgc ttaaggatcg gttgtcgagt aaggatctcc     2161 aggcatcaaa taaaacgaaa ggctcagtcg aaagactggg cctttcgttt tatctgttgt     2221 ttgtcggtga acgctctcta ctagagtcac actggctcac cttcgggtgg gcctttctgc     2281 gtttatagga tcctaactcg agcctaggga tatattccgc ttcctcgctc actgactcgc     2341 tacgctcggt cgttcgactg cggcgagcgg aaatggctta cgaacggggc ggagatttcc     2401 tggaagatgc caggaagata cttaacaggg aagtgagagg gccgcggcaa agccgttttt     2461 ccataggctc cgcccccctg acaagcatca cgaaatctga cgctcaaatc agtggtggcg     2521 aaacccgaca ggactataaa gataccaggc gtttccccct ggcggctccc tcgtgcgctc     2581 tcctgttcct gcctttcggt ttaccggtgt cattccgctg ttatggccgc gtttgtctca     2641 ttccacgcct gacactcagt tccgggtagg cagttcgctc caagctggac tgtatgcacg     2701 aaccccccgt tcagtccgac cgctgcgcct tatccggtaa ctatcgtctt gagtccaacc     2761 cggaaagaca tgcaaaagca ccactggcag cagccactgg taattgattt agaggagtta     2821 gtcttgaagt catgcgccgg ttaaggctaa actgaaagga caagttttgg tgactgcgct     2881 cctccaagcc agttacctcg gttcaaagag ttggtagctc agagaacctt cgaaaaaccg     2941 ccctgcaagg cggttttttc gttttcagag caagagatta cgcgcagacc aaaacgatct     3001 caagaagatc atcttattaa//
